# Discovery of Nanosota-9 as anti-Omicron nanobody therapeutic candidate

**DOI:** 10.1371/journal.ppat.1012726

**Published:** 2024-11-26

**Authors:** Gang Ye, Fan Bu, Divyasha Saxena, Hailey Turner-Hubbard, Morgan Herbst, Benjamin Spiller, Brian E. Wadzinski, Lanying Du, Bin Liu, Jian Zheng, Fang Li

**Affiliations:** 1 Department of Pharmacology, University of Minnesota Medical School, Minneapolis, Minnesota, United States of America; 2 Center for Emerging Viruses, University of Minnesota, Minneapolis, Minnesota, United States of America; 3 Center for Predictive Medicine, University of Louisville, Kentucky, United States of America; 4 Department of Pharmacology, Vanderbilt University School of Medicine, Nashville, Tennessee, United States of America; 5 Institute for Biomedical Sciences, Georgia State University, Atlanta, Georgia, United States of America; 6 Hormel Institute, University of Minnesota, Austin, Minnesota, United States of America; 7 Department of Microbiology and Immunology, University of Louisville, Kentucky, United States of America; Loyola University Chicago Stritch School of Medicine, UNITED STATES OF AMERICA

## Abstract

Omicron subvariants of SARS-CoV-2 continue to pose a significant global health threat. Nanobodies, single-domain antibodies derived from camelids, are promising therapeutic tools against pandemic viruses due to their favorable properties. In this study, we identified a novel nanobody, Nanosota-9, which demonstrates high potency against a wide range of Omicron subvariants both in vitro and in a mouse model. Cryo-EM data revealed that Nanosota-9 neutralizes Omicron through a unique mechanism: two Nanosota-9 molecules crosslink two receptor-binding domains (RBDs) of the trimeric Omicron spike protein, preventing the RBDs from binding to the ACE2 receptor. This mechanism explains its strong anti-Omicron potency. Additionally, the Nanosota-9 binding epitopes on the spike protein are relatively conserved among Omicron subvariants, contributing to its broad anti-Omicron spectrum. Combined with our recently developed structure-guided in vitro evolution approach for nanobodies, Nanosota-9 has the potential to serve as the foundation for a superior anti-Omicron therapeutic.

## Author summary

Omicron continues to evolve and spread among humans, making the development of effective and broad-spectrum treatments a top priority for global health. Nanobodies, which are single-domain antibodies derived from camelids, show great promise due to their small size and ability to target conserved, hidden regions on the virus. In this study, we identified a novel nanobody called Nanosota-9, which effectively inhibits most major Omicron subvariants. Our cryo-EM analysis revealed a unique structural mechanism by which Nanosota-9 neutralizes Omicron. Like many other anti-SARS-CoV-2 antibodies and nanobodies, Nanosota-9 binds to the receptor-binding domain (RBD) of Omicron spike proteins, blocking receptor binding. However, unlike others, two Nanosota-9 molecules crosslink two RBDs of the trimeric spike, enhancing its anti-Omicron potency. One of the two binding sites on the RBD that Nanosota-9 targets significantly overlaps with the receptor-binding site and is relatively conserved, while the other site is fully conserved, expanding its anti-Omicron spectrum. Conventional antibodies cannot access the same binding sites on the spike protein as Nanosota-9, highlighting its unique neutralization mechanism. Combined with our newly developed structure-guided in vitro evolution approach for nanobodies, Nanosota-9 has strong potential as a superior anti-Omicron therapeutic.

## Introduction

Omicron subvariants of SARS-CoV-2 continue to circulate among human populations, posing a threat to global health. Since Omicron’s emergence in the fall of 2021, new Omicron subvariants have continually appeared, from the earliest BA.1 to later BA.5, and more recent ones like XBB.1.5 and JN.1, up to the current KP.2 and KP.3 [1,2]. It is likely that Omicron will persist in human populations long-term, necessitating ongoing interventions, including vaccines and antiviral therapeutics. Although SARS-CoV-2 vaccines are available, breakthrough infections are common [3,4]. While several anti-SARS-CoV-2 therapeutics exist, Paxlovid is the most effective and widely used. Paxlovid is a combination of a protease inhibitor and a helper drug that inhibits liver function, raising concerns about potential drug-drug interactions and liver and kidney toxicity [5,6]. Moreover, relying on a single drug increases the risk of viral escape mutations [7]. Therefore, novel anti-Omicron therapeutics are urgently needed to effectively control Omicron.

Antibodies are among the primary antiviral therapeutics due to their high target specificity and hence high safety [8]. The SARS-CoV-2 spike protein is the top target for antibody therapeutics [9,10]. It is a homotrimer on the virus surface, consisting of three copies of the receptor-binding S1 subunit and a trimeric membrane-fusion S2 stalk. During viral entry, the receptor-binding domain (RBD) in S1 binds to its host receptor ACE2 on the cell surface, the spike protein is cleaved by host proteases at the S1/S2 boundary, and S2 undergoes a dramatic structural change to fuse the viral and host membranes. Early in the pandemic, we discovered three key structural mechanisms of the SARS-CoV-2 spike protein: its RBD has a high affinity for human ACE2, its RBD can either stand up for receptor binding or lie down for immune evasion, and it can be cleaved by the human protease furin [11,12]. Because the RBD induces most of the neutralizing immune responses in humans, it is heavily targeted by antibody therapeutics. The RBD contains two subdomains: a core structure and a receptor-binding motif (RBM), with the RBM mediating receptor binding [10,13]. RBD-targeting antibody therapeutics need to bind to the RBM with very high affinity, blocking ACE2 binding. Ideally, they should also target both the standing up and lying down RBDs to counter viral evasion. Indeed, the RBD is the primary target of most neutralizing antibody responses [14]. However, human antibody therapeutics have faced setbacks against SARS-CoV-2 due to the extensive evolution of the RBD [15,16]. Currently, there are no published antibody treatments for the latest KP.2 and KP.3 Omicron subvariants. Importantly, because of their large size, human antibodies cannot easily access conserved cryptic epitopes [17,18], limiting their potency and antiviral spectrum. Small-sized antibodies are potential solutions as anti-SARS-CoV-2 entry inhibitors.

Nanobodies are single-domain antibodies derived from camelid animals [19–21]. Due to their small size, they possess excellent therapeutic properties as antiviral drugs. They have superior epitope accessibility and tissue permeability, which contribute to their high potency. They can access conserved hidden epitopes, providing them with broad antiviral spectrums. Nanobodies are easy to produce, transport, and store, making them cost-effective. They can potentially be administered intranasally, offering an attractive needle-free therapy option [22,23]. Additionally, they demonstrate minimal toxicity and immunogenicity in humans [24,25]. A nanobody drug is clinically available to treat a blood clotting disorder [26], validating the safety of nanobodies as human therapeutics. Numerous nanobody inhibitors have been developed against pre-Omicron variants and early Omicron subvariants [21]. However, due to the extensive evolution of Omicron, there is an urgent need for novel nanobody inhibitors against recent and current Omicron subvariants to counter the spread of Omicron. So far, only one study has identified a nanobody inhibitor against the JN.1 subvariant, but it was only tested *in vitro*, without any *in vivo* or structural data [27]. No nanobody has been developed against the KP.2 or KP.3 subvariant. Therefore, broad-spectrum nanobody inhibitors against recent and current Omicron subvariants with demonstrated *in vivo* efficacy are yet to be discovered.

Previously, we developed several nanobody inhibitors, collectively named the Nanosota series, against the prototypic SARS-CoV-2 spike [28–30]. More recently, we introduced a novel structure-guided in vitro evolution approach to rapidly adapt nanobodies to emerging SARS-CoV-2 variants [31]. In this study, we discovered a novel nanobody, named Nanosota-9, from an alpaca immunized with the Omicron BA.5 spike protein. Nanosota-9 exhibits high anti-Omicron potency and a broad anti-Omicron spectrum. It not only possesses many of the ideal qualities mentioned above, but also utilizes a unique structural mechanism to inhibit Omicron. Combined with our structure-guided in vitro evolution approach, Nanosota-9 holds great potential as the foundation for a superior anti-Omicron therapeutic.

## Results

### Discovery of Nanosota-9 from immunized alpaca

To discover anti-Omicron nanobodies, we immunized an alpaca with the recombinant spike ectodomain of the Omicron subvariant BA.5. We collected peripheral blood mononuclear cells (PBMCs) from the immunized alpaca and established an induced nanobody phage display library. We then screened this library using the recombinant BA.5 spike ectodomain as bait. Through this process, we discovered a nanobody named Nanosota-9. We constructed a human Fc-tagged version of Nanosota-9, named Nanosota-9-Fc. As previously shown, compared to monomeric nanobodies, Fc-tagged nanobodies have significantly enhanced *in vivo* half-lives due to surpassing the size threshold for kidney clearance [29]. Nevertheless, the sizes of Fc-tagged nanobodies are still half those of human antibodies. Importantly, Fc-tagged nanobodies maintain the single-domain structure for target binding, which is crucial for binding cryptic epitopes. Therefore, Nanosota-9-Fc was used in this study to evaluate its anti-Omicron activities, while Nanosota-9-His (His-tagged Nanosota-9) was used only for structural studies.

**Fig 1:**
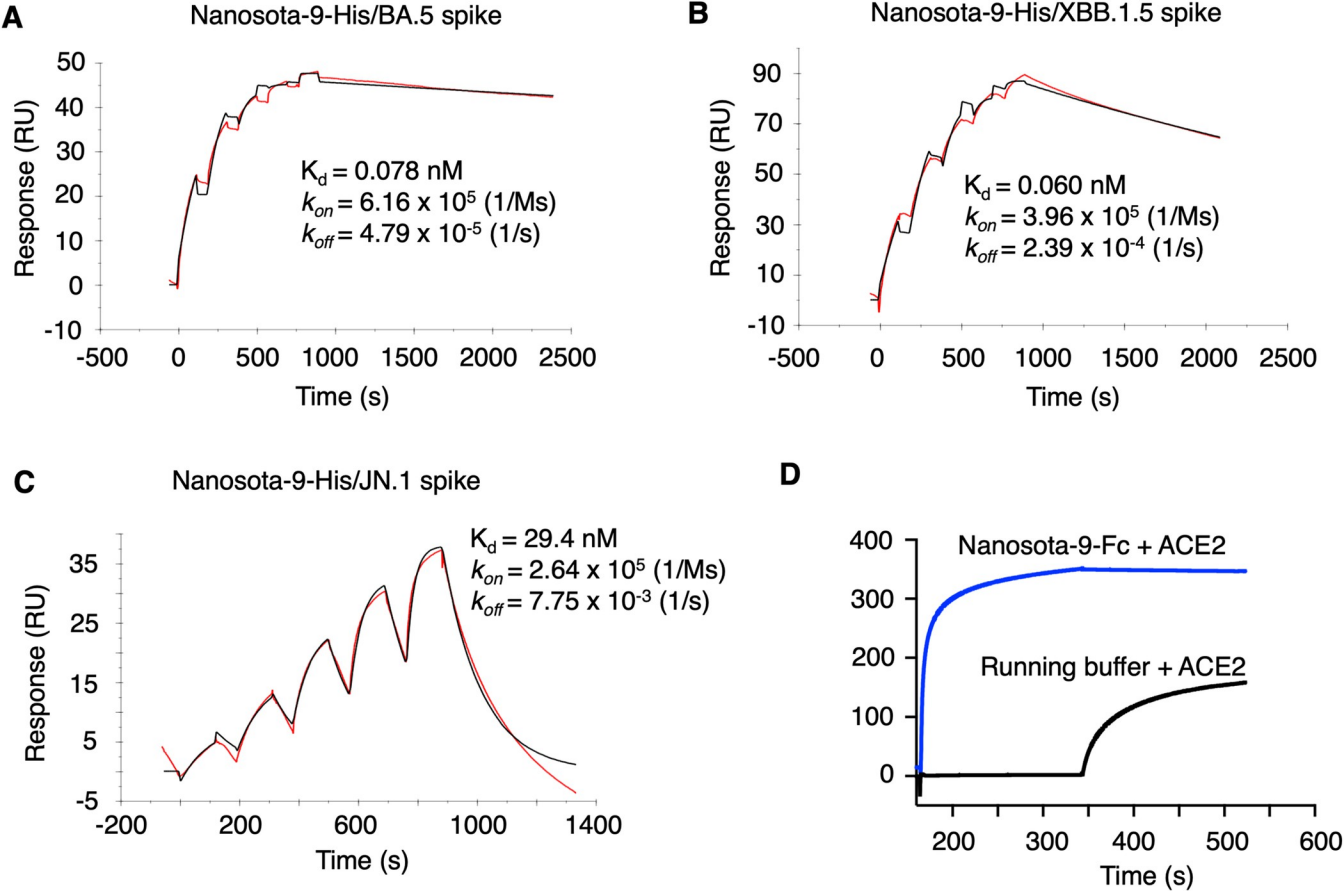
Binding kinetics between Nanosota-9 and Omicron spike ectodomains as measured by surface plasmon resonance (SPR). **(A)-(C)** Each of the recombinant spike ectodomains from Omicron subvariants (BA.5, XBB.1.5, and JN.1) was immobilized on a CM5 sensor chip. His-tagged Nanosota-9 (Nanosota-9-His) was injected at different concentrations. The resulting data were analyzed using Biacore Evaluation Software. **(D)** Competition SPR analysis. The JN.1 spike ectodomain was immobilized on two CM5 sensor chips. Fc-tagged Nanosota-9 (Nanosota-9-Fc) was injected onto the first chip, while the second chip received only the running buffer. Subsequently, a mixture of recombinant human ACE2 and Nanosota-9-Fc was added to the first chip, and only ACE2 was added to the second chip. Sensorgrams from both chips were overlaid for comparison. The lack of change in the resonance signal after ACE2 injection from the Nanosota-9-Fc-bound JN.1 spike ectodomain indicated that ACE2 could not displace Nanosota-9-Fc from binding to the JN.1 spike ectodomain.

We characterized the binding of Nanosota-9-Fc to its targets through several assays. First, we conducted an ELISA to evaluate the binding of Nanosota-9-Fc to recombinant spike ectodomains from different Omicron subvariants. The results showed that Nanosota-9-Fc binds with high affinity to the spike ectodomains from the BA.5, XBB.1.5, and JN.1 subvariants, but does not bind to the spike ectodomain from the BA.1 subvariant (S1 Fig). Second, we used surface plasmon resonance (SPR) to measure the binding affinity between Nanosota-9-His and the spike ectodomains from the BA.5, XBB.1.5, and JN.1 subvariants (Fig 1A, 1B and 1C). The results indicated that Nanosota-9-His exhibits tight binding to all three spike ectodomains, with dissociation constants (Kds) of 0.078 nM, 0.060 nM, and 29.4 nM for the BA.5, XBB.1.5, and JN.1 spikes, respectively. Third, we carried out competitive SPR to investigate potential competition between Nanosota-9 and ACE2 for binding to the XBB.1.5 spike ectodomain (Fig 1D). The results showed that Nanosota-9 and ACE2 do not bind the XBB.1.5 spike protein simultaneously. Together, these data indicate that Nanosota-9 potently targets the spikes of BA.5, XBB.1.5, and JN.1, but not BA.1, and that Nanosota-9 binds to overlapping epitopes on the spikes with ACE2.

### Neutralizing potency of Nanosota-9 against Omicron

**Fig 2:**
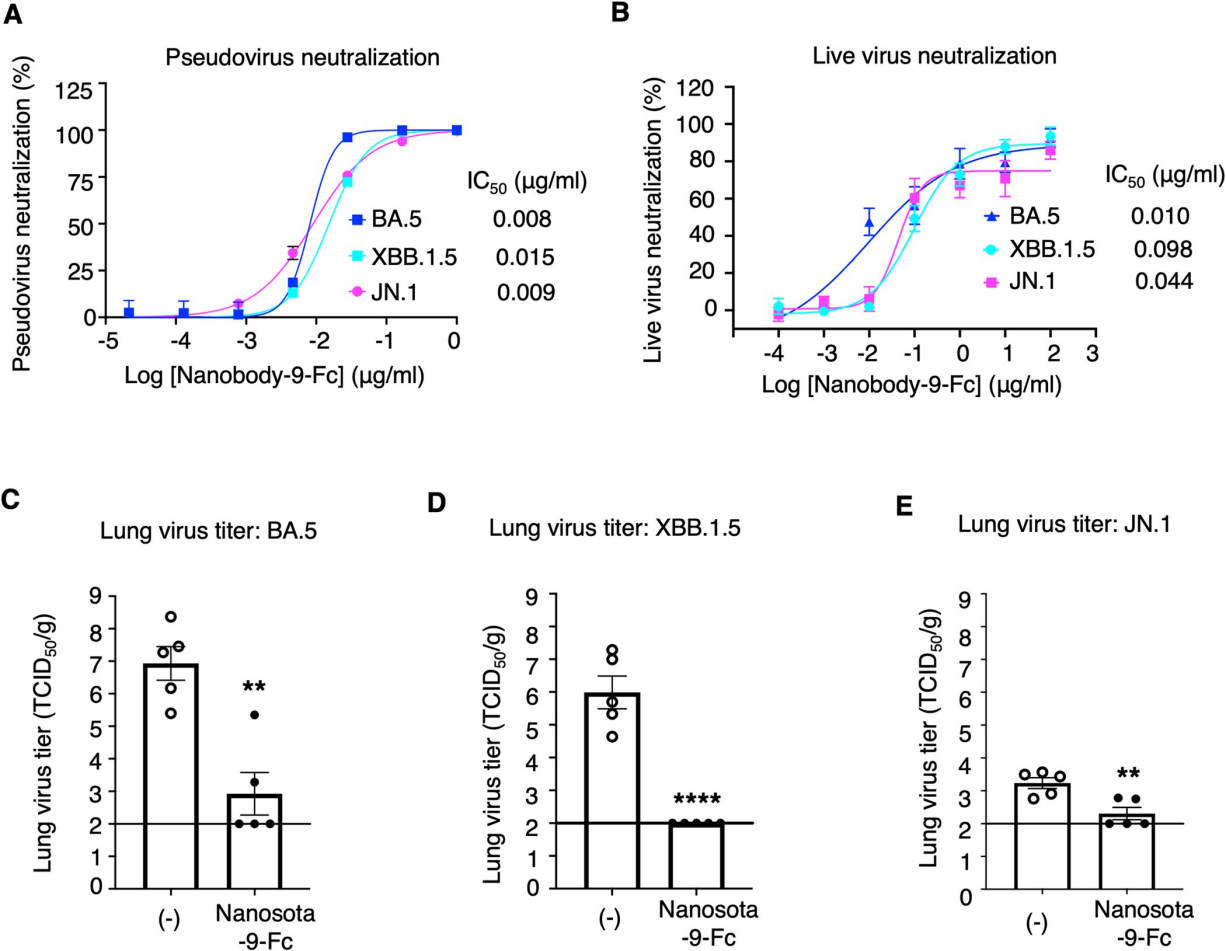
Neutralization potency of Nanosota-9 against Omicron subvariants *in vitro* and *in vivo*. **(A)** Efficacy of Nanosota-9-Fc in neutralizing Omicron pseudoviruses. Retroviruses pseudotyped with each of the Omicron spike proteins (BA.5, XBB.1.5, and JN.1) were used to enter human ACE2-expressing cells in the presence of Nanosota-9-Fc at different concentrations. Entry efficiency was measured by the luciferase signal accompanying entry. Nonlinear regression was performed using a log (inhibitor) versus response curve. The efficacy of the nanobody was expressed as the concentration capable of neutralizing pseudovirus entry by 50% (i.e., IC_50_). Data are the mean ± SEM (n = 3). **(B)** Efficacy of Nanosota-9-Fc in neutralizing live infectious Omicron *in vitro*. Each of the Omicron subvariants infected permissive Vero cells in the presence of Nanosota-9-Fc at different concentrations. Cell viability following 96 hours of incubation was determined using Neutral Red assay (Sigma-Aldrich). Nonlinear regression was performed using a log (inhibitor) versus response curve. The efficacy of Nanosota-9-Fc against each of the Omicron subvariants was calculated and expressed as the concentration capable of reducing the virus induced Cytopathic effect (CPE) by 50% (IC_50_) compared to control serum-exposed virus. Data are the mean ± SEM (n = 4). **(C)-(E)** Efficacy of Nanosota-9-Fc in neutralizing live infectious Omicron in mice. Nanosota-9-Fc was administered at a dosage of 10 mg/kg body weight and 4 hours post-challenge. Mice were challenged via intranasal inoculation with each of the Omicron subvariants. In the treatment group (n = 5), mice received Nanosota-9-Fc via intraperitoneal injection. In the control group (n = 5), mice were administered PBS buffer. Virus titers in the mouse lungs on day 2 post-challenge were measured. The detection limit of the lung virus titer measurements is indicated by a line. Comparisons of lung virus titers between the control and treatment groups were performed using an unpaired two-tailed Student’s *t*-test. Error bars represent SEM. **p<0.01; ****p<0.0001.

We evaluated the neutralizing potency of Nanosota-9 against Omicron using two different *in vitro* assays. First, we conducted an Omicron pseudovirus entry assay. Lentiviruses pseudotyped with each of the Omicron spikes (i.e., Omicron pseudoviruses) were used to enter human ACE2-expressing cells in the presence of Nanosota-9-Fc. The results showed that Nanosota-9-Fc potently neutralized the entry of all three Omicron pseudoviruses, BA.5, XBB.1.5, and JN.1, with IC_50_ values of 8 ng/ml, 15 ng/ml, and 9 ng/ml, respectively (Fig 2A). Second, we performed a live Omicron infection assay, where each of the live Omicron viruses was used to infect ACE2-expressing cells in the presence of Nanosota-9-Fc. The results showed that Nanosota-9-Fc potently neutralized the infection of all three Omicron subvariants, BA.5, XBB.1.5, and JN.1, with IC_50_ values of 10 ng/ml, 98 ng/ml, and 44 ng/ml, respectively (Fig 2B). Thus, Nanosota-9-Fc is a potent neutralizer of all three Omicron subvariants *in vitro*.

We also evaluated the neutralizing potency of Nanosota-9 against Omicron in vivo. Mice were challenged with each of the three Omicron subvariants, BA.5, XBB.1.5, and JN.1, through intranasal inoculation. For each subvariant, the mice were divided into two groups: four hours post-challenge, one group received Nanosota-9-Fc at a dosage of 10 mg/kg via the intraperitoneal route, while the other group received PBS buffer. All six groups of mice were monitored for lung virus titers two days post-challenge. The results showed that both the BA.5 and XBB.1.5 subvariants replicated efficiently in the mice, reaching titers of 10^7^ TCID_50_ and 10^6^ TCID_50_ in the lungs on day 2, respectively (Fig 2C and 2D). Nanosota-9-Fc reduced the titers of both BA.5 and XBB.1.5 in the lungs by 10,000-fold, with the XBB.1.5 titer reduced to near the detection limit (Fig 2C and 2D). However, the JN.1 subvariant replicated inefficiently in the mice, reaching a titer of only 10^3^ TCID_50_ in the lungs on day 2 (Fig 2E), suggesting that JN.1 differs from BA.5 and XBB.1.5 in their replication efficiency in the current mouse model. Nevertheless, Nanosota-9-Fc significantly reduced the JN.1 titer in the lungs to near the detection limit (Fig 2E). Therefore, Nanosota-9-Fc is a potent neutralizer of all three Omicron subvariants *in vivo*.

### Structural basis for anti-Omicron efficacy of Nanosota-9

**Fig 3:**
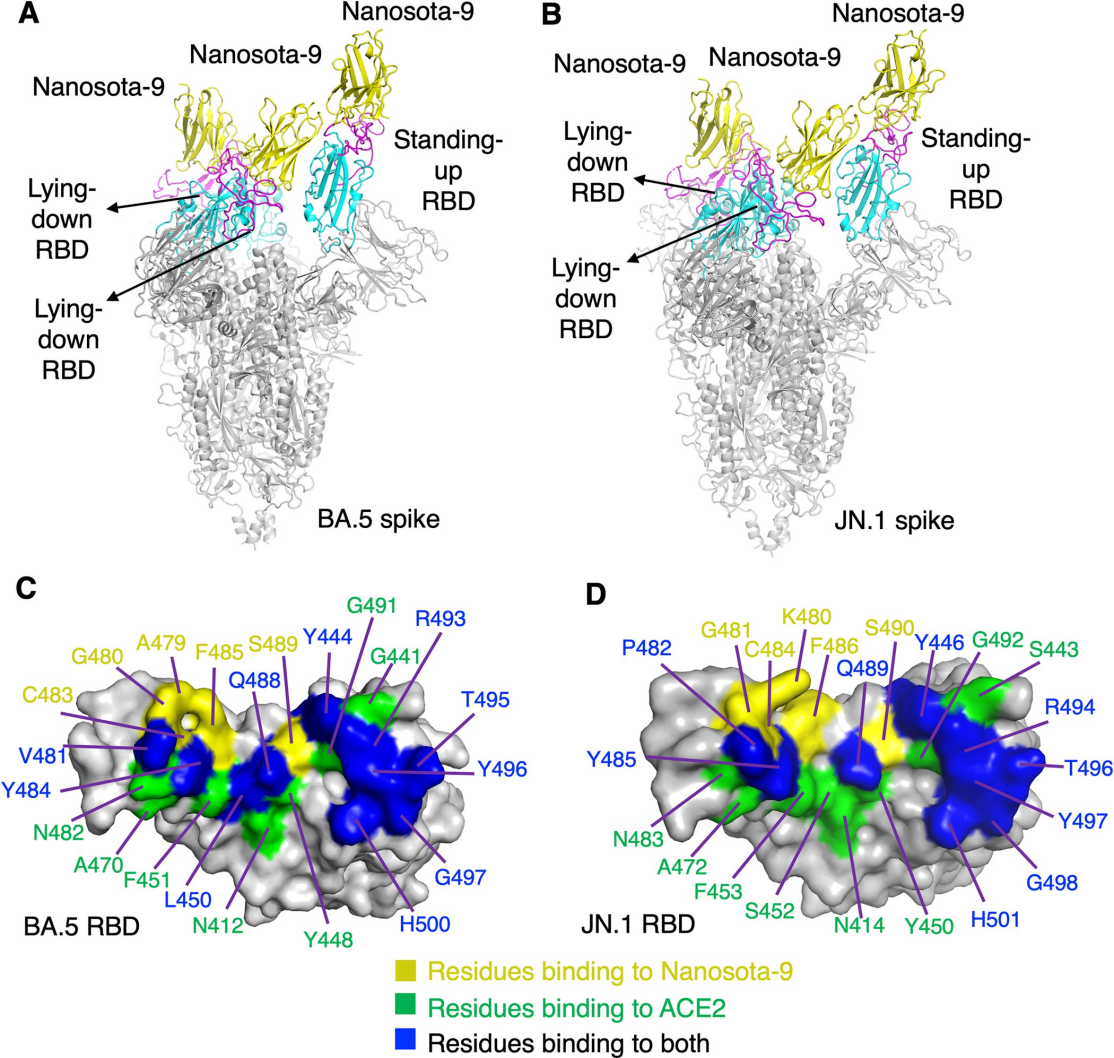
Overall structures of Omicron spike ectodomains complexed with Nanosota-9. **(A)** Cryo-EM structures of BA.5 spike ectodomain complexed with Nanosota-9. Three spike-bound Nanosota-9 molecules are colored in yellow. The three RBDs are colored in cyan (core region) and magenta (RBM), with one RBD in the standing up position and the other two in the lying down position. **(B)** Cryo-EM structures of JN.1 spike ectodomain complexed with Nanosota-9. **(C)** Footprints of Nanosota-9 and ACE2 on the BA.5 RBD. Nanosota-9-binding RBD residues, ACE2-binding RBD residues, and dual-binding RBD residues are colored as indicated. The Nanosota-9 epitope heavily overlaps with the ACE2-binding site on the RBD, blocking receptor binding to the RBD and thereby neutralizing Omicron entry. **(C)** Footprints of Nanosota-9 and ACE2 on the JN.1 RBD.

**Fig 4:**
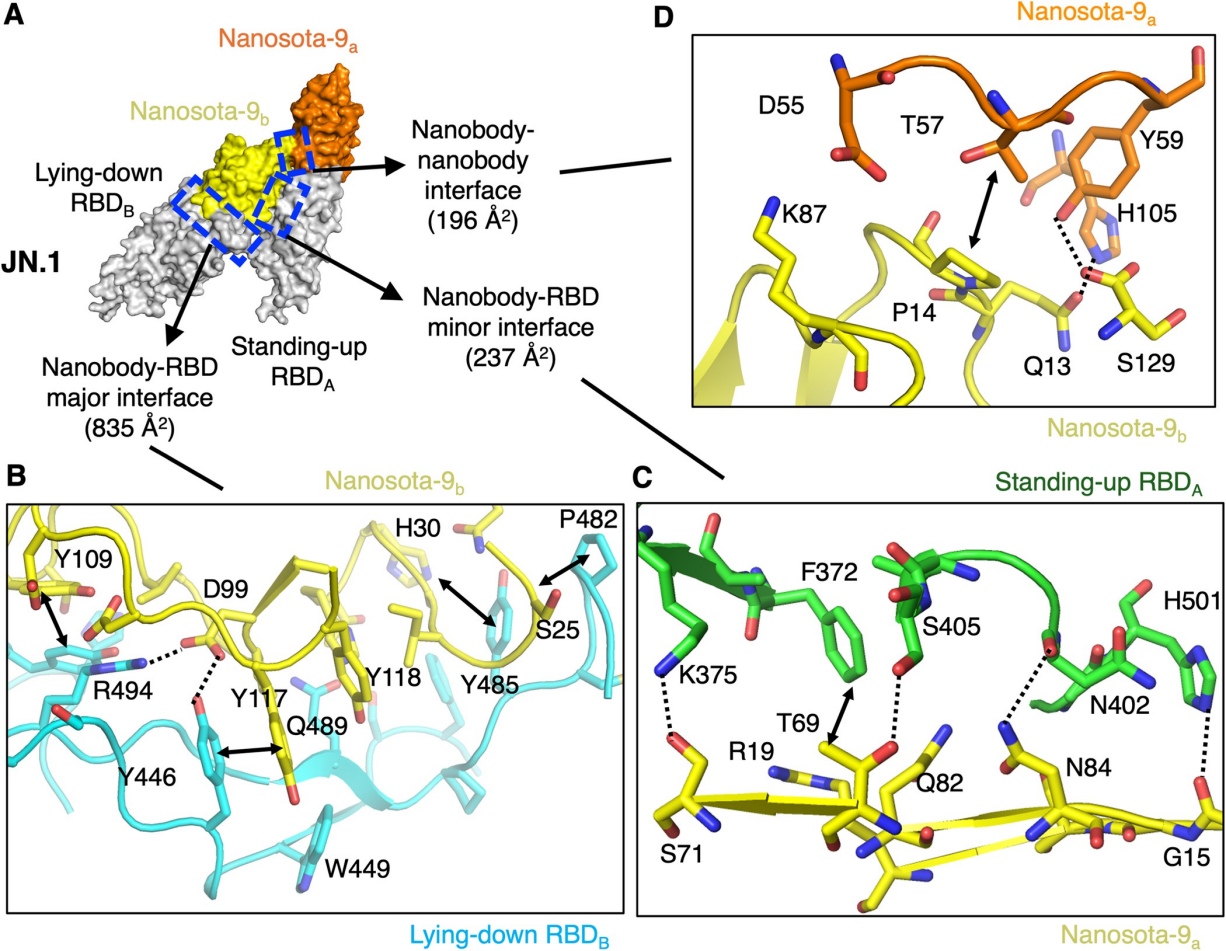
Nanosota-9 binds to Omicron spike proteins using a novel crosslinking mechanism. **(A)** Two Nanosota-9 molecules crosslink two RBDs together. The 2:2 binding mode creates three interfaces: a major interface between Nanosota-9 and the RBM of one RBD, a minor interface between Nanosota-9 and the core of another RBD, and an additional interface between the two Nanosota-9 molecules. **(B)** Detailed interactions at the major interface between Nanosota-9 and the RBM of one RBD. **(C)** Detailed interactions at the minor interface between Nanosota-9 and the core of another RBD. **(D)** Detailed interactions at the additional interface between the two Nanosota-9 molecules. Dotted lines indicate hydrogen bonds or salt bridges. Double arrows indicate hydrophobic stacking interactions. The crosslinking mechanisms and the three interfaces stabilize the lying down RBD, preventing it from binding to the ACE2 receptor and enhancing Nanosota-9’s anti-Omicron potency.

To understand the structural basis of Nanosota-9’s potency against Omicron, we determined the cryo-EM structures of the BA.5 and JN.1 spike ectodomains complexed with Nanosota-9 (Figs 3A, 3B, S2, S3, S4 and S1 Table). The structures revealed that Nanosota-9 binds to both spikes in a similar manner: it engages all three copies of the RBD, with one in a “standing-up” conformation and the other two in a “lying-down” conformation. The structural interfaces between Nanosota-9 and the BA.5 and JN.1 RBDs are also highly similar (Figs 3C, 3D, 4, and S5). Nanosota-9 binds to the same epitope on the RBDs, which significantly overlaps with the ACE2 binding site (Fig 3C and 3D). Specifically, Nanosota-9 directly contacts 15 BA.5 RBD residues, while ACE2 directly contacts 17 BA.5 RBD residues, with 10 residues shared between both. This result aligns with the competitive SPR findings, which showed that Nanosota-9 and ACE2 cannot bind to the spike protein simultaneously (Fig 1D). Therefore, similar to Nanosota-1, -2, -3, and -4, Nanosota-9 neutralizes viral entry by blocking the spike protein’s receptor binding.

Nanosota-9 exhibits a unique binding mode with Omicron spikes. While three Nanosota-9 molecules bind to each trimeric spike (which has three RBDs), their distribution is asymmetrical: two of the three Nanosota-9 molecules crosslink two RBDs, connecting one standing-up RBD with one lying-down RBD. This contrasts with Nanosota-1, -2, -3, and -4, each of which binds to an RBD in a one-to-one manner. The 2:2 binding mode of Nanosota-9 with the RBD creates three distinct interfaces (Figs 4A, S5A, and S6). The major interface between Nanosota-9 and the RBM buries 835 Å^2^ of surface area and is dominated by strong hydrophobic stacking interactions (Figs 4B and S5B). The minor interface involves the RBD core, burying 237 Å^2^ primarily through hydrogen bonds (Figs 4C and S5C). Additionally, the interface between two Nanosota-9 molecules buries 196 Å^2^, featuring fewer interactions than the other two interfaces (Figs 4D and S5D). Due to the relatively small sizes of the latter two interfaces, the 2:2 binding mode of Nanosota-9 with the RBD may only occur in the context of the trimeric spike. Collectively, these three interfaces stabilize the Nanosota-9/spike complex by locking one RBD in the "lying-down" position and the other in the "standing-up" position, rendering both RBDs’ ACE2-binding sites inaccessible. This unique binding mode enhances Nanosota-9’s anti-Omicron potency.

### Anti-Omicron spectrum of Nanosota-9

**Fig 5:**
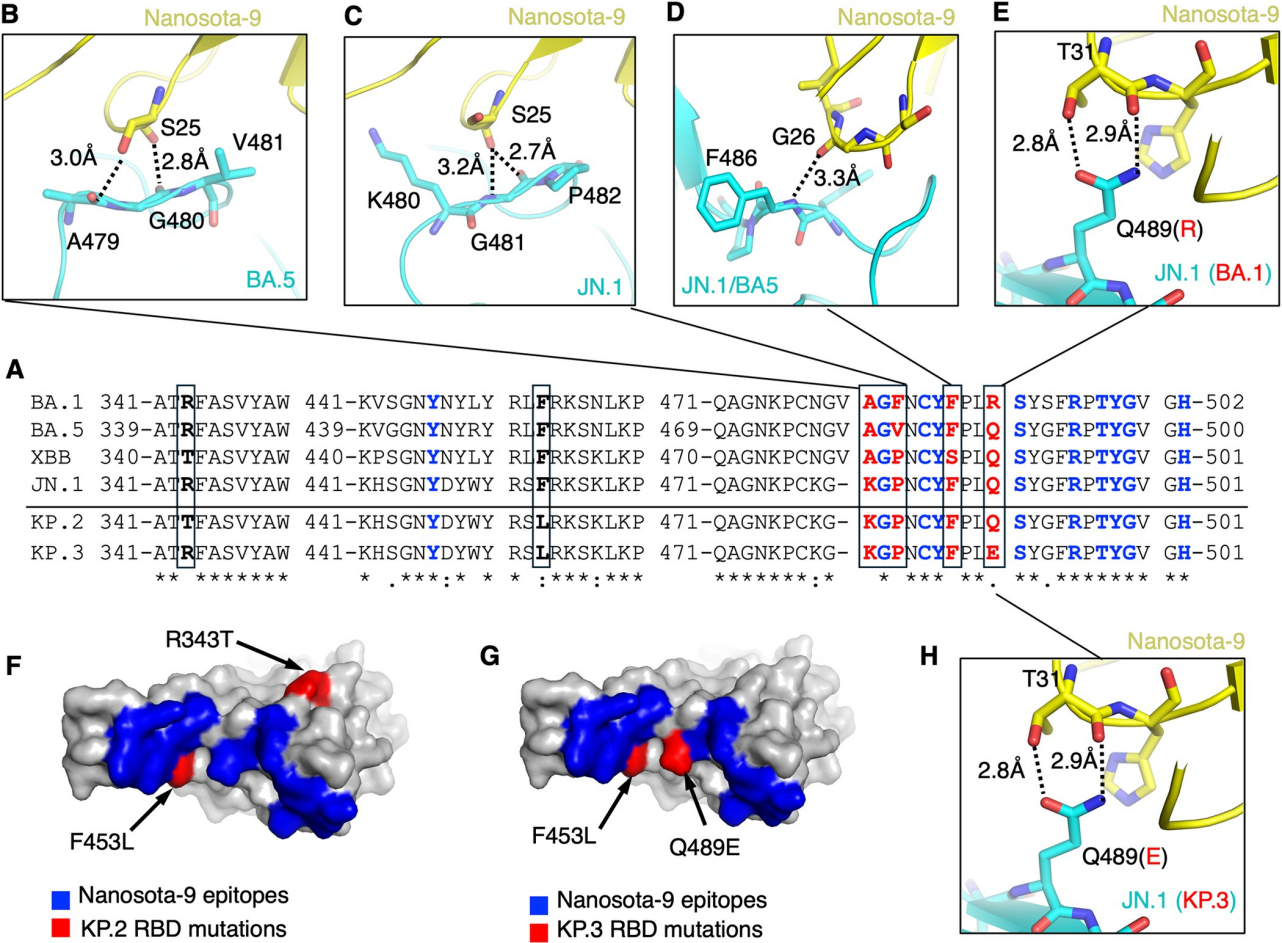
Evolution of Omicron RBDs within the Nanosota-9 binding epitopes. **(A)** Sequence alignment of the Nanosota-9-binding RBD residues among six Omicron subvariants: BA.1, BA.5, XBB.1.5, JN.1, KP.2, and KP.3. RBD residues in direct contact with Nanosota-9 are colored in blue (conserved among Omicron subvariants) or red (mutated among Omicron subvariants). Two RBD residues that are not in direct contact with Nanosota-9 but underwent mutations in KP.2 and KP.3 subvariants are labeled in bold black. Mutated residues among Omicron subvariants are boxed. Asterisks indicate positions with a single, fully conserved residue. Colons indicate positions with strongly conserved residues. Periods indicate positions with weakly conserved residues. **(B)-(D)** Structural details of Nanosota-9-binding RBD residues that underwent mutations among the three Omicron subvariants, BA.5, XBB.1.5, and JN.1. **(E)** Structural details of Nanosota-9-binding RBD residue 489 that underwent a mutation in Omicron subvariant BA.1. **(F)-(G)** Mapping of RBD residues that underwent mutations in Omicron subvariants KP.2 and KP.3. **(H)** Structural details of Nanosota-9-binding RBD residue 489 that underwent a mutation in Omicron subvariant KP.3.

To understand the anti-Omicron spectrum of Nanosota-9, we analyzed the evolution of Omicron subvariants at the Nanosota-9-binding interfaces. Sequence alignment of the RBDs from BA.5, XBB.1.5, and JN.1 revealed three mutations in residues that interact directly with Nanosota-9: 478, 480, and 486 (Fig 5A). However, these residues all interact through their main-chain functional groups, so side-chain changes likely have little effect on the binding of Nanosota-9 (Fig 5B, 5C and 5D). This explains why Nanosota-9 effectively neutralizes BA.5, XBB.1.5, and JN.1. Furthermore, only one residue in contact with Nanosota-9 mutated between BA.1 and the other subvariants: residue 489 (Fig 5E). In the JN.1 RBD, Gln489 forms two hydrogen bonds with Thr31 of Nanosota-9, involving both the side-chain hydroxyl group and the main-chain carbonyl oxygen of Thr31 (Fig 5E). The Q489R mutation likely disrupts these favorable interactions and introduces clashes between Arg489’s long side chain and Nanosota-9, explaining Nanosota-9’s inability to neutralize BA.1. These structural insights align with biochemical and virological findings on Nanosota-9’s antiviral effectiveness against earlier and recent Omicron subvariants.

We extended this analysis to two currently circulating subvariants, KP.2 and KP.3. Sequence alignment of the RBDs from JN.1, KP.2, and KP.3 revealed two mutations in KP.2 (residues 343 and 453) and two in KP.3 (residues 453 and 489) (Fig 5A, 5F and 5G). Residues 343 and 453 do not interact directly with Nanosota-9, so these changes likely do not affect its binding to KP.2 RBD. However, in the KP.3 RBD, the Q489E mutation likely maintains a hydrogen bond with Thr31’s side chain but introduces an unfavorable interaction between the negatively charged side chain of Glu489 and the partially negatively charged main-chain carbonyl oxygen of Thr31 (Fig 5H). As a result, similar to BA.1, Nanosota-9 likely fails to neutralize KP.3 due to the altered residue at position 489. With detailed structural data now available for the RBD/Nanosota-9 interfaces, our newly developed structure-guided in vitro evolution approach can help evolve Nanosota-9 to overcome the Q489E mutation [31], potentially enabling neutralization of KP.3.

**Fig 6:**
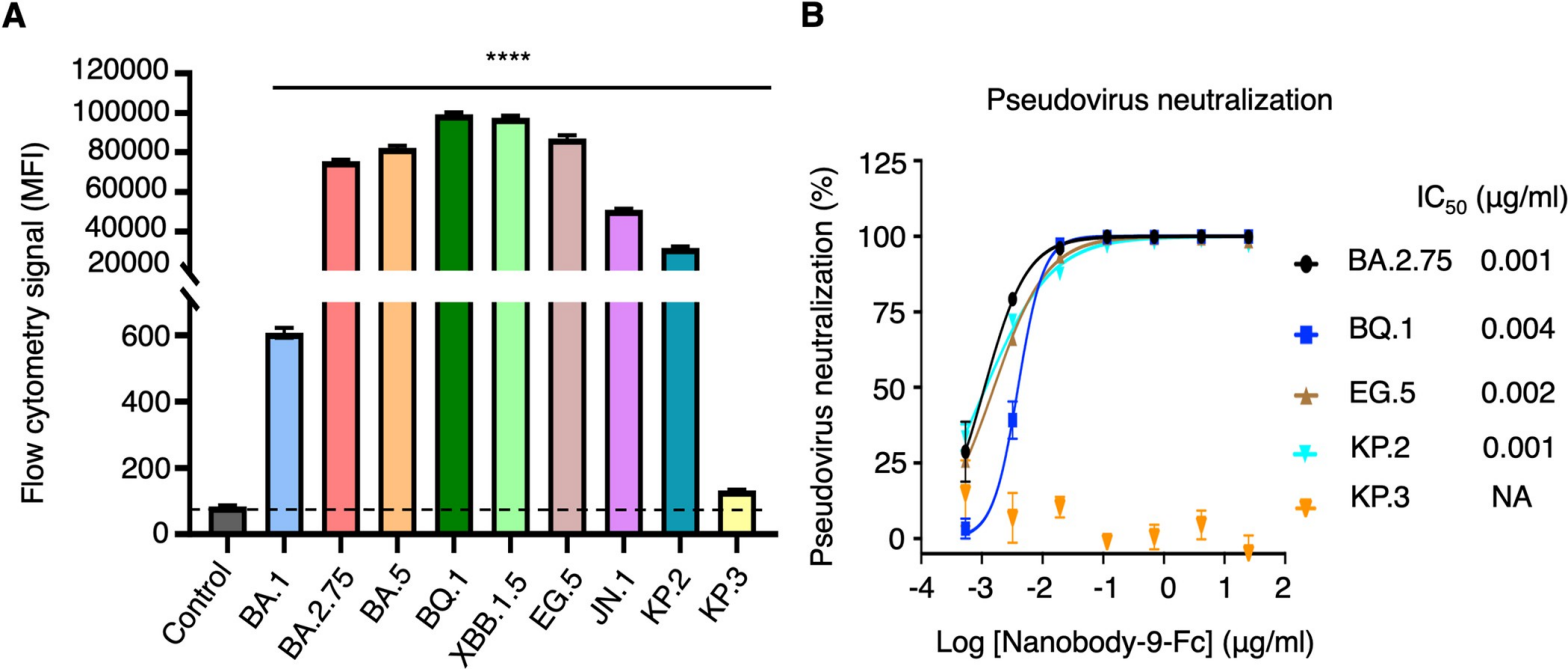
Biochemical assessment of the anti-Omicron spectrum of Nanosota-9. **(A)** Flow cytometry analysis of the interaction between Nanosota-9-Fc and cell surface-expressed spike proteins from major Omicron subvariants. The flow cytometry signal, expressed as mean fluorescence intensity (MFI), was measured using PE anti-Fc tag antibodies, which target the Fc-tagged Nanosota-9 protein bound to spike-positive cells. The control group consisted of cells transfected with an empty vector. Data are presented as mean ± SEM (n = 3). Statistical differences between the control group and each experimental group were analyzed using a two-tailed Student’s *t*-test. ****p < 0.0001. (B) Neutralization efficacy of Nanosota-9-Fc against pseudoviruses of major Omicron subvariants. The assay was conducted as described in Fig 2A.

To validate the above structural analysis, we performed two biochemical assays to extensively assess Nanosota-9’s anti-Omicron spectrum. First, using flow cytometry, we tested Nanosota-9’s binding to cell-surface-expressed spike proteins from nine Omicron subvariants: BA.1, BA.2.75, BA.5, BQ.1, XBB.1.5, EG.5, JN.1, KP.2, and KP.3. The results showed high-affinity binding to all subvariants except BA.1 and KP.3 (Figs 6A and S7). Next, we conducted a pseudovirus neutralization assay to examine Nanosota-9’s potency against the same subvariants (except BA.5, XBB.1.5, and JN.1, which were measured earlier; Fig 2A). Again, Nanosota-9 neutralized all except BA.1 and KP.3 with high potency (Fig 6B). Thus, the biochemical data corroborate the structural analysis, indicating that Nanosota-9 has broad anti-Omicron coverage, except for BA.1 and KP.3, likely due to the mutation at position 489 in their RBDs. Structure-guided in vitro evolution of Nanosota-9 can help overcome this mutation and further expand its anti-Omicron spectrum.

## Discussion

Current intervention strategies against Omicron have significant limitations, underscoring the urgent need for novel inhibitors. Due to the constant evolution of Omicron, all existing entry inhibitors have struggled to keep pace: currently, there are no published antibodies or nanobody inhibitors effective against the current KP.2 and KP.3 subvariants. Although the RBD has the highest mutation rate compared to other regions of the spike protein, RBD-targeting antibodies remain the most dominant and potent compared to those targeting other spike regions [14]. We recently developed a structure-guided in vitro evolution approach to rapidly adapt nanobodies to emerging viral variants [31], offering hope that entry inhibitors can keep up with Omicron’s evolution. In this study, we identified a novel nanobody, Nanosota-9, which targets a relatively conserved epitope on the Omicron RBD and effectively neutralizes the majority of main Omicron subvariants. With its unique mechanism, potent neutralization, and broad spectrum, Nanosota-9 stands out as a promising therapeutic candidate against Omicron, especially when combined with the new nanobody evolution strategy.

The neutralizing potency of Nanosota-9 against Omicron stems from its unique structural mechanisms in binding to the Omicron spike proteins. First, Nanosota-9’s primary binding site on the RBD heavily overlaps with the ACE2-binding site, suggesting that Nanosota-9 neutralizes Omicron by blocking receptor binding [12]. Second, Nanosota-9 binds to both the “standing up” and “lying down” RBDs, effectively countering viral evasion [11]. Third, two Nanosota-9 molecules crosslink two RBDs together, locking one of the two RBDs in the “lying down” position, preventing it from binding to the ACE2 receptor and enhancing the anti-Omicron potency of Nanosota-9. Docking a human antibody into the same binding pocket as Nanosota-9 revealed clashes between the human antibody and the spike protein (S8A Fig), indicating that this binding site is not accessible to human antibodies. Furthermore, comparing the Nanosota-9 epitope with those of human antibodies targeting the Omicron RBD revealed that only one human antibody shares an overlapping epitope (S8B Fig). However, if this human antibody were to bind to a lying-down RBD, it would clash with a standing-up RBD (S8B Fig), confirming that the Nanosota-9 binding site is inaccessible to human antibodies. Hence, this crosslinking strategy represents a unique antiviral mechanism for nanobodies. Comparison of the epitopes of Nanosota-9 to those of Nanosota-2, -3, and -4 reveals that the Nanosota-9 epitopes overlap with but also differ from those of Nanosota-2 and -3 (S9 Fig), which explains why only Nanosota-9, but not the other nanobodies, can crosslink the RBDs. These structural mechanisms collectively explain the neutralizing potency of Nanosota-9 against Omicron.

The broad spectrum of Nanosota-9 against Omicron is due to the structural features of the interfaces that it forms with Omicron RBDs. Nanosota-9 forms two binding interfaces with the RBD. The minor interface with the core of the RBD is completely conserved among recent and current Omicron subvariants likely due to its inaccessibility to human antibodies. The major interface with the RBM of the RBD heavily overlaps with the ACE2-binding site, restricting the virus’s ability to mutate many of the dual binding RBD residues. Consequently, the major epitope is relatively conserved among recent and current Omicron subvariants. Although a few mutations have occurred at the major epitope among different Omicron subvariants, detailed structural analysis suggests that they are unlikely to significantly impact the RBDs’ binding to Nanosota-9. The relative conservation of the major epitope is also likely due to its inaccessibility to human antibodies when the RBD is in the lying-down position. However, since the major epitope remains accessible to human antibodies when the RBD is in the standing-up position, it is not fully conserved. As demonstrated in this study, while Nanosota-9 effectively neutralizes most major Omicron subvariants, a single mutation at residue 489 likely reduced its potency against BA.1 and KP.3. Despite this, the restricted accessibility to human antibodies helps maintain the relative conservation of the major epitope. Moreover, if a limited number of mutations occur within this epitope, our newly developed structure-guided in vitro evolution approach can help adapt Nanosota-9 to keep pace with the evolving Omicron subvariants at this site.

In summary, Nanosota-9 possesses several properties that make it an ideal anti-Omicron therapeutic candidate. It directly blocks ACE2 binding to the RBD and neutralizes viral entry. By binding to both “standing-up” and “lying-down” RBDs, it counteracts viral evasion. Nanosota-9 employs a unique crosslinking mechanism to further inhibit ACE2 binding and enhance antiviral potency. The interfaces it forms with the RBD are relatively conserved among Omicron subvariants. Beyond these structural features, our previous research has identified other advantageous properties of nanobodies that are likely applicable to Nanosota-9. For example, nanobodies’ high expression yield and in vitro thermostability make them cost-effective [29], and their potential for intranasal administration offers an appealing needle-free therapy option [30], in addition to traditional injections. Our earlier studies also demonstrated that nanobodies are significantly more effective when used prophylactically than therapeutically [29, 30]. Therefore, while Nanosota-9 was only tested therapeutically in mice in this study, its high therapeutic potency suggests it could also be effective as a prophylactic treatment. Overall, combined with our recently developed structure-guided in vitro evolution method, Nanosota-9 has the potential to become a superior anti-Omicron therapeutic.

## Methods

### Ethics statement

This study was performed in strict accordance with the recommendations in the Guide for the Care and Use of Laboratory Animals of the National Institutes of Health. All of the animals were handled according to approved institutional animal care and use committee (IACUC) protocols of the University of Louisville (protocol number: 22134).

### Cell lines, plasmids and viruses

HEK293T cells (American Type Culture Collection (ATCC)) were grown in Dulbecco’s modified Eagle medium (DMEM) (containing 10% fetal bovine serum, 2 mM L-glutamine, 100 units/mL penicillin, and 100 μg/mL streptomycin). 293F cells (ThermoFisher) were grown in FreeStyle 293 Expression Medium (ThermoFisher). Vero E6 cells (ATCC) were cultured in Eagle’s minimal essential medium (EMEM) (containing 100 units/ml penicillin, 100 μg/ml streptomycin, and 10% fetal bovine serum). ss320 *E*. *coli* (Lucigen) and TG1 *E*. *coli* (Lucigen) were grown in 2YT medium. All mammalian cells were authenticated by ATCC using STR profiling and were also tested for mycoplasma contamination. No commonly misidentified cell lines were used.

Mutations were introduced to the original SARS-CoV-2 spike gene [12] to generate the spike genes from the following Omicron subvariants: BA.1 subvariant (GISAID: EPI_ISL_6590782.2), BA.5 subvariant (GISAID: EPI_ISL_12954165), XBB.1.5 subvariant (GISAID: EPI_ISL_17774216), EG.5 subvariant (GISAID: EPI_ISL_17524442), JN.1 subvariant (GISAID: EPI_ISL_17774216), KP.2 subvariant (GISAID: EPI_ISL_19214303), and KP.3 subvariant (GISAID: EPI_ISL_19214243). The spike genes from the BA.2.75 subvariant (GISAID: EPI_ISL_13502529) and BQ.1 subvariant (GISAID: EPI_ISL_16609492) were synthesized (GenScript). Each of the spike genes was cloned into the pcDNA3.1(+) vector with a C-terminal C9-tag sequence.

Genes encoding Omicron spike ectodomains (residues 1 to 1208 for BA.1, 1 to 1206 for BA.5, 1 to 1207 for XBB.1.5, and 1 to 1207 for JN.1) were each subcloned into Lenti-CMV vector (Vigene Biosciences) with a C-terminal foldon trimerization tag sequence following a His tag sequence. For the spike ectodomain construct, a D614G mutation, two mutations in the furin cleavage site (from RRAR to AGAR), and six proline mutations were introduced to the S2 subunit region to stabilize the spike ectodomains in their prefusion state [32, 33].

A plasmid encoding Fc-tagged Nanosota-9 (Nanosota-9-Fc) was constructed into Lenti-CMV vector with an N-terminal tissue plasminogen activator (tPA) signal peptide sequence and a C-terminal human IgG_1_ Fc tag sequence.

A gene encoding monomeric Nanosota-9 (Nanosota-9-His) was cloned into PADL22c vector (Antibody Design Labs) with an N-terminal PelB leader sequence and C-terminal His tag and HA tag sequences.

The Omicron viruses (hCoV-19/USA/COR-22-063113/2022 for BA.5, hCoV-19/USA/MD-HP40900/2022 for XBB.1.5, and hCoV-19/USA/New York/PV96109/2023 for JN.1) were obtained through BEI Resources, NIH. Experiments involving live infectious Omicron viruses were conducted at the University of Louisville in approved biosafety level 3 laboratories.

### Construction of induced nanobody phage display library

An induced nanobody phage display library was constructed as described previously [30,34]. Briefly, an alpaca was immunized six times with 125 μg of purified BA.5 spike ectodomain in Gerbu adjuvant. After immunization, blood was collected, and peripheral blood mononuclear cells (PBMCs) were isolated using Sepmate centrifugal devices according to the manufacturer’s protocol (Stemcell Technologies). A cDNA library was then constructed through reverse transcription using oligo dT primers and Superscript IV reverse transcriptase (ThermoFisher). A nested PCR strategy was employed to amplify the coding regions of the nanobody fragments. The resulting PCR products were cloned into a modified pADL22 vector (Antibody Design Labs), and the phage library was generated following the manufacturer’s protocol (Antibody Design Labs). The final library contained 8 x 10^8^ colonies. Ten randomly selected clones from the library were sequenced, each showing a unique sequence.

### Screening of induced nanobody phage display library

To identify anti-Omicron nanobodies, the above nanobody phage display library was used for bio-panning as previously described [29, 30]. 5 μg of purified BA.5 spike ectodomain in 500 μl of PBS buffer was coated onto an immuno tube (ThermoFisher) and incubated overnight at 4°C. The tube was then blocked with 2% milk in PBS buffer for 2 hours. After blocking, the nanobody phage library was added and incubated for 1 hour with gentle shaking. The tube was washed five times each with PBST (PBS with tween-20) and PBS buffers. After washing, the retained phages were eluted using 500 μl of 100 mM triethylamine, neutralized with 250 μl of 1 M Tris-HCl (pH 7.5), and then used to infect log-phase ss320 E. coli. Single colonies were picked into 96-well plates containing 2YT A+ medium and grown overnight at 37°C. The ss320 cultures were then transferred into fresh 2YT medium and grown for 3 hours at 37°C. Nanobody expression was induced with 1 mM IPTG. The supernatants were screened by ELISA against the BA.5 spike ectodomain to identify strong binders. One of the binders, which neutralized BA.5 pseudovirus entry, was named Nanosota-9 and further evaluated.

### Protein expression and purification

Nanosota-9-His was expressed and purified from bacteria as previously described [29,30]. Briefly, the nanobody was extracted from the periplasm of ss320 E. coli following induction with 1 mM IPTG. The E. coli cells were collected, resuspended in 15 ml of TES buffer (0.2 M Tris pH 8, 0.5 mM EDTA, 0.5 M sucrose), and shaken on ice for 1 hour. The suspension was then diluted with 40 ml of ¼ TES buffer and shaken on ice for another hour. The protein in the supernatant was sequentially purified using a Ni-NTA column and a Superdex200 gel filtration column (Cytiva).

Omicron spike ectodomains (with a C-terminal His tag) and Nanosota-9-Fc were produced in 293F mammalian cells as previously described [30,35]. Briefly, lentiviral particles were generated using a plasmid encoding one of these proteins, which were then used to infect 293F cells for the selection of stable cell lines in the presence of Puromycin (Gibco). Proteins were harvested from the supernatants of the cell culture medium and purified using a Ni-NTA column for His-tagged spike ectodomains or a Protein A column for Nanosota-9-Fc. Further purification was performed using a Superose 6 Increase 10/300 gel filtration column (Cytiva) for spike ectodomains and a Superdex 200 gel filtration column (Cytiva) for Nanosota-9-Fc.

### ELISA

To detect the binding between His-tagged Omicron spike ectodomains and HA-tagged nanobodies, an ELISA was performed as previously described [29,30]. Briefly, ELISA plates were coated with one of the recombinant Omicron spike ectodomains (100 ng at 2 μg/ml) and incubated overnight at 4°C, followed by blocking with 2% BSA in PBS buffer. After three washes with PBST, the plates were incubated with the supernatant from ss320 E. coli containing one of the nanobodies for 1 hour. After another three washes with PBST buffer, the plates were incubated with an HRP-conjugated anti-HA antibody (1:1,000) (Sigma-Aldrich) for 1 hour. The plates were washed again three times with PBST buffer, and 50 μl of ELISA substrate (Invitrogen) was added. The reactions were stopped by adding 10 μl of 1N H₂SO₄. The absorbance at 450 nm (A450) was measured using a Synergy LX Multi-Mode Reader (BioTek).

To detect the binding between His-tagged Omicron spike ectodomains and Nanosota-9-Fc, an HRP-conjugated anti-human Fc antibody (1:3000) (Jackson ImmunoResearch) was used instead of the anti-HA antibody. All other procedures remained the same as described above.

### Surface plasmon resonance (SPR)

To measure the binding affinity between the Omicron spike ectodomains and Nanosota-9-His, a surface plasmon resonance (SPR) assay was conducted using a Biacore S200 system (Cytiva) as previously described [29,30]. Briefly, each Omicron spike ectodomain was immobilized on a CM5 sensor chip (Cytiva) through chemical crosslinking. Serial dilutions of Nanosota-9-His were injected at different concentrations (8 nM, 16 nM, 32 nM, 64 nM, and 128 nM) using a running buffer composed of 10 mM HEPES, 150 mM NaCl, and 0.05% Tween 20. The resulting data were analyzed using Biacore Evaluation Software (Cytiva).

To assess the potential competition between human ACE2 and Nanosota-9-Fc for binding to the Omicron spike protein, a competition SPR experiment was performed as previously described [30]. The JN.1 spike ectodomain (with a His tag) was immobilized onto two CM5 sensor chips (Cytiva) with 800 resonance units (RUs) for each chip. Nanosota-9-Fc (5 μM) was then injected onto the first sensor chip, while running buffer was injected onto the second chip as a control. After saturating the first chip with Nanosota-9-Fc, a mixture of recombinant human ACE2 (His-tagged, at 5 μM) and Nanosota-9-Fc (5 μM) was injected onto the first chip. In the control chip, only ACE2 was injected. The resulting sensorgrams from both chips were overlaid, using the first injection as the baseline. The competitive binding of human ACE2 and Nanosota-9-Fc to the JN.1 spike ectodomain was evaluated by comparing the SPR binding signals from the mixed nanobody/ACE2 injections with those from the ACE2-only injection.

### Flow cytometry assay

A flow cytometry assay was performed to evaluate the binding affinity between Nanosota-9-Fc and the spike protein from each Omicron subvariant, as previously described [17,36]. Briefly, 2 μg of each full-length spike-expressing plasmid with a C-terminal C9 tag sequence was transfected into HEK293T cells in 6-well plates. 24 hours after transfection, cells were detached using Accutase solution (Sigma-Aldrich), washed twice with cold PBS buffer, and resuspended in 500 μl of cold PBS buffer. The cells were fixed with 1 ml of 4% paraformaldehyde (ThermoFisher) and incubated at room temperature for 20 minutes. They were then washed three times with 1% BSA and resuspended in 500 μl of 1% BSA. Nanosota-9-Fc was added to a final concentration of 10 μg/ml and incubated at room temperature for 30 minutes. The cells were washed three times with 1% BSA and resuspended in 200 μl of 1% BSA. Subsequently, 2 μl of anti-C9 antibody and 5 μl of PE anti-human IgG Fc antibody (BioLegend) were added to the cells and incubated on ice in the dark for 30 minutes. The cells were then washed three times with cold PBS buffer and resuspended in 1 ml of cold PBS buffer. Data were acquired using a BD LSRII cytometer and analyzed with FlowJo software (version 10).

### Pseudovirus entry assay

The neutralizing potency of Nanosota-9-Fc against Omicron pseudoviruses was assessed using a pseudovirus entry assay as previously described [30,35]. Briefly, HEK293T cells were co-transfected with a pcDNA3.1(+) plasmid encoding an Omicron spike protein, a helper plasmid psPAX2, and a reporter plasmid plenti-CMV-luc to produce pseudoviruses. The pseudoviruses were harvested 72 hours post-transfection, incubated with nanobodies at varying concentrations at 37°C for 30 minutes, and then used to transduce HEK293T cells stably expressing human ACE2 in 96-well plates. After 60 hours, the medium was removed, and 80 μl of lysis buffer (Promega) was added to the plates. The cells were lysed with gentle shaking for 25 minutes, and 30 μl of the cell lysate was transferred to 96-well OptiPlates (Revvity Health Sciences). Luciferase substrate (30 μl; Promega) was added to each well, and Relative Light Units (RLUs) were measured using an EnSpire plate reader (PerkinElmer). The efficacy of the nanobody against each Omicron pseudovirus was expressed as the nanobody concentration required to inhibit pseudovirus entry by 50% (IC_50_).

### SARS-CoV-2 microneutralization assay

The neutralizing potency of Nanosota-9-Fc against live Omicron infections was assessed using a virus microneutralization assay as previously described [37]. Briefly, Nanosota-9-Fc was serially diluted tenfold in DMEM, starting at 100 μg/ml. Each dilution was prepared in quadruplicate and mixed with one of the Omicron subvariants—BA.5, XBB.1.5, or JN.1 (0.001 MOI)—at 37°C for 45 minutes. The mixtures were then added to Vero E6 cells overexpressing ACE2 and TMPRSS2 (A2T2) in a 96-well plate that had been cultured overnight. After 1 hour, the virus/Nanosota-9-Fc mixtures were replaced with 1X DMEM supplemented with 5% FBS. Cell viability was determined after 96 hours of incubation using a Neutral Red assay (Sigma-Aldrich). The efficacy of Nanosota-9-Fc against each Omicron subvariant was expressed as the concentration needed to reduce the virus-induced cytopathic effect (CPE) by 50% (IC_50_) compared to the control serum-exposed virus.

### Omicron challenge experiment in a mouse model

The neutralizing potency of Nanosota-9-Fc against infectious Omicron in vivo was assessed using Omicron challenge experiments in a mouse model as previously described [29,30]. Briefly, female C57BL/6J mice (n = 5 per group) were challenged via intranasal inoculation with one of the Omicron subvariants (10^4^ PFU per mouse) in a 50 μl volume of DMEM. Infected mice received either Nanosota-9-Fc (10 mg/kg body weight) or PBS via intraperitoneal injection 4 hours post-challenge. Mice were euthanized on day 2 post-infection, and the collected lung tissues were homogenized and stored at -80°C until further analysis. Viral titers in the lung tissues were determined by TCID_50_ assay as previously described [37]. Briefly, Vero E6 cells were seeded into 96-well tissue culture plates and incubated overnight at 37°C. The next day, lung homogenate supernatants were serially diluted tenfold in viral growth medium (DMEM containing 5% FBS) and added in quadruplicates to the cell-seeded plates. The plates were incubated at 37°C in a humidified incubator with 5% CO_2_. After 4 days post-infection, cells were fixed in 10% neutral-buffered formalin and stained with 0.1% crystal violet to observe for cytopathic effect (CPE). The TCID_50_ dose was calculated using the Reed and Muench method.

### Cryo-EM grid preparation and data acquisition

The complexes of BA.5 spike ectodomains and Nanosota-9-His (4 μl at 0.8 μM) and of JN.1 spike ectodomain and Nanosota-9-His (4 μl at 0.8 μM) were applied to freshly glow-discharged Quantifoil R1.2/1.3 300-mesh copper grids (EM Sciences), blotted for 4 seconds at 22°C under 100% chamber humidity, and plunge-frozen in liquid ethane using a Vitrobot Mark IV (FEI). Cryo-EM data were collected using EPU (ThermoFisher) equipped with a K3 direct electron detector and a BioQuantum energy filter (Gatan) in CDS mode. Movies were collected at a nominal magnification of 130,000x (corresponding to 0.664 Å per pixel, a slit width of 20 eV, and a nominal defocus value between −1.0 to −2.0 μm. Statistics of cryo-EM data collection are summarized in S1 Table.

### Cryo-EM data processing, model building and refinement

Cryo-EM data were processed using cryoSPARC v4.5.1 [38], with the procedure detailed in S2 and S3 Figs. Briefly, dose-fractionated movies underwent Patch motion correction with MotionCor2 [39] and Patch CTF estimation with CTFFIND-4.1.13 [40]. Particles were picked using both Blob picker and Template picker in cryoSPARC v4.5.1, and duplicates were removed with the Remove Duplicate Particles Tool. Junk particles were eliminated through multiple rounds of 2D classifications. Particles from the high-quality 2D classes were used for Ab-initio Reconstruction of four maps. These initial models served as starting references for heterogeneous refinement (3D classification). The high-quality 3D classes were further refined using non-uniform, and CTF refinements to generate the final maps. Particles in the good 3D class were imported into RELION-4.0 [41] using the csparc2star.py module (UCSF pyem v0.5. Zenodo) and subjected to signal subtraction to keep only two receptor-binding subunits of the spike and two Nanosota-9 molecules. Particles with the subtracted signal (RBD/Nanosota-9) were then subjected to local refinements to improve densities in cryoSPARC v4.5.1. Map resolutions were determined by gold-standard Fourier shell correlation (FSC) at 0.143 between the two half-maps. Local resolution variation was estimated from the two half-maps in cryoSPARC v4.5.1.

Initial model building of the spike/nanobody complexes was performed in Coot-0.8.9 [42] using PDB 8IOS as the starting models for the BA.5 and JN.1 spike ectodomains, respectively. The initial model of each nanobody was predicted using SWISS-MODEL (https://swissmodel.expasy.org/) and then fitted into the density map. Several rounds of refinement in Phenix-1.16 [43] and manual building in Coot-0.8.9 were performed until the final reliable models were obtained. Model and map statistics are summarized in S1 Table. Figures were generated using UCSF Chimera X v0.93 [44] and PyMol v2.5.2 [45].

## Supporting information

S1 Fig Binding interactions between Nanosota-9-Fc and Omicron spike ectodomains as measured by ELISA. ELISA plates were coated with one of the recombinant Omicron spike ectodomains and then incubated with Nanosota-9-Fc. Spike-bound Nanosota-9-Fc was detected using anti-human Fc antibody.
(TIF)

S2 Fig Flow chart of cryo-EM image processing and 3D reconstruction for the complex of BA.5 spike ectodomain and Nanosota-9. Representative raw cryo-EM image and 2D classes are presented. 3D refinements using all the particles from good 3D classes generated a 3.01 Å map. Further local refinement improved the density for the bound nanobody. The angular distribution plot, final maps, half-map FSC curves and accompanying local resolution illustrations are enclosed in the dashed black boxes.
(PDF)

S3 Fig Flow chart of cryo-EM image processing and 3D reconstruction for the complex of JN.1 spike ectodomain and Nanosota-9. Representative raw cryo-EM image and 2D classes are presented. 3D refinements using all the particles from good 3D classes generated a 2.99 Å map. Further local refinement improved the density for the bound nanobody. The angular distribution plot, final maps, half-map FSC curves and accompanying local resolution illustrations are enclosed in the dashed black boxes.
(PDF)

S4 Fig Cryo-EM densities of the three interfaces formed among two Nanosota-9 molecules and two Omicron RBDs. These three interfaces are: the major interface between the standing-up RBD and Nanosota-9, the minor interface between the lying-down RBD and Nanosota-9, and the additional interface between two Nanosota-9 molecules.
(TIF)

S5 Fig Detailed interactions between the BA.5 spike ectodomain and Nanosota-9. This figure was prepared in the same way as Fig 4, except that the BA.5 spike ectodomain was used instead of the JN.1 spike ectodomain.
(TIF)

S6 Fig Surface presentation of the JN.1 spike ectodomain complexed with Nanosota-9. **(A)** Side view of the structure. **(B)** Top view of the structure.
(TIF)

S7 Fig Flow cytometry data showing the interactions between Nanosota-9-Fc and cell surface-expressed spike proteins from major Omicron subvariants. (TIF)

S8 Fig The Nanosota-9 binding site is inaccessible to human antibodies when the RBD is in a lying-down position. **(A)** Docking of a human antibody to the Nanosota-9 binding site on the Omicron spike. The antigen-binding domains of a human antibody (PDB 7B3O) were docked onto the structure of the JN.1 spike ectodomain/Nanosota-9 complex by structurally aligning the heavy-chain (HC) antigen-binding domain of the human antibody and Nanosota-9. Nanobodies and heavy-chain antigen-binding domains of human antibodies are evolutionarily and functionally related. The blue circle indicates a clash between the light-chain (LC) antigen-binding domain of the human antibody and the lying-down RBD, suggesting that human antibodies cannot access the Nanosota-9 binding site on Omicron spikes. **(B)** Overlay of the structures of human antibodies and Nanosota-9 all bound to the Omicron RBD. The structures of the Omicron spike ectodomains complexed with Fabs from human antibodies (PDB IDs labeled in parentheses) were overlaid with the JN.1 spike ectodomain/Nanosota-9 complex by structurally aligning their spike RBDs. Only one human antibody (PDB 7TAT) shares an overlapping epitope with Nanosota-9 on the lying-down RBD, but it clashes with a standing-up RBD, as indicated by the blue circle. Structural alignments were performed using PyMol.
(TIF)

S9 Fig Overlay of the structures of four nanobodies on the SARS-CoV-2 RBD. The structures of the prototypic SARS-CoV-2 spike each complexed with Nanosota-2, -3, or -4 (PDBs: 8G72, 8G74, and 8G75) were overlaid with the structure of the JN.1 spike complexed with Nanosota-9 through structural alignment of their spike RBDs. Nanosota-9 clashes with Nanosota-2 and -3, but not with Nanosota-4. Structural alignments were performed using PyMol.
(TIF)

S1 Table Cryo-EM data collection, refinement and validation statistics of the spike Nanosota-9 complexes. (PDF)
