# Supplementary figures and images for: Discovery of Nanosota-9 as anti-Omicron nanobody therapeutic candidate
Source: PLoS Pathog. 2024 Nov 26;20(11):e1012726. doi: 10.1371/journal.ppat.1012726 (PMC11630572; doi:10.1371/journal.ppat.1012726)

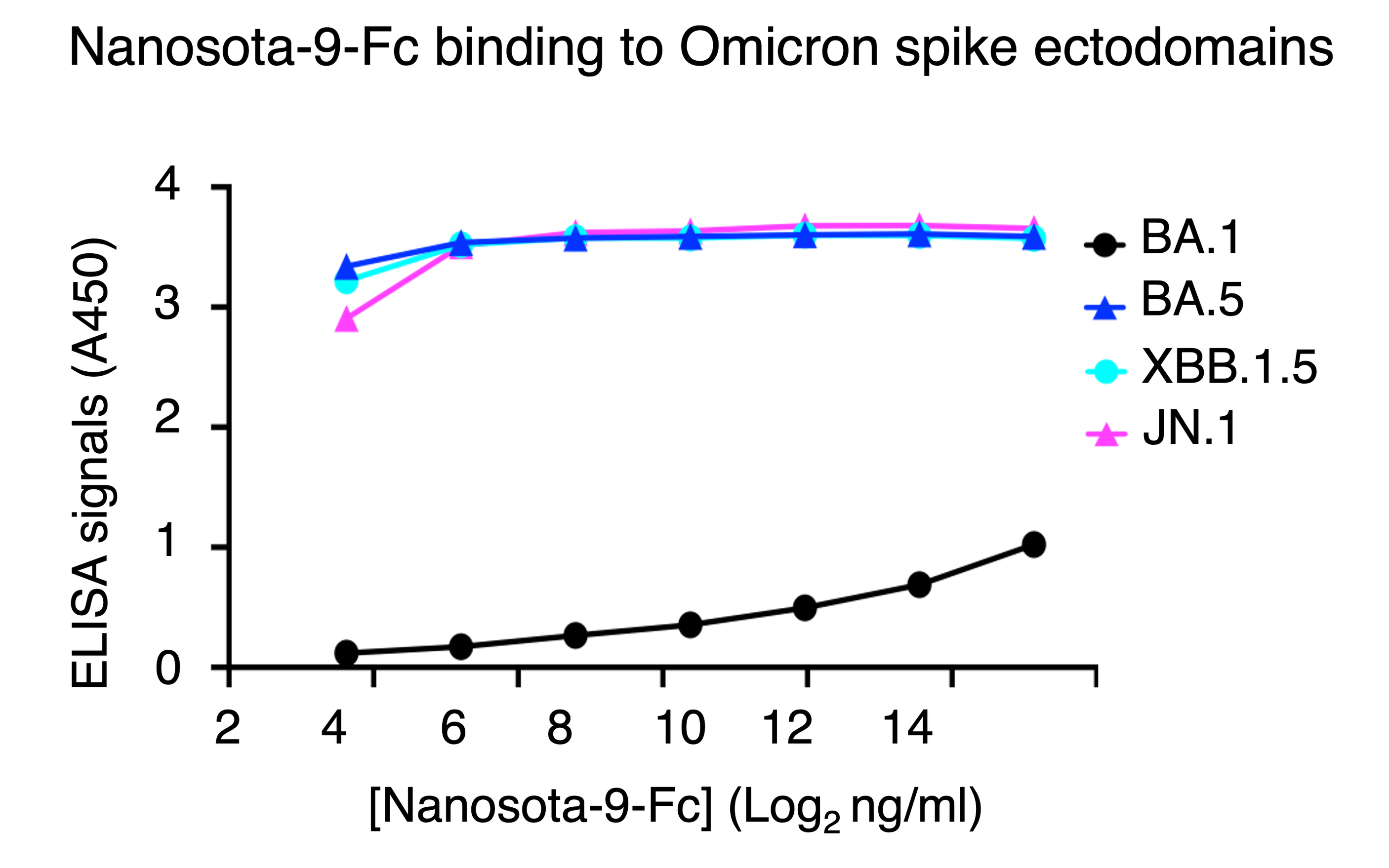

Supplement: S1 Fig — ELISA plates were coated with one of the recombinant Omicron spike ectodomains and then incubated with Nanosota-9-Fc. Spike-bound Nanosota-9-Fc was detected using anti-human Fc antibody. (TIF) [file ppat.1012726.s001.tif]

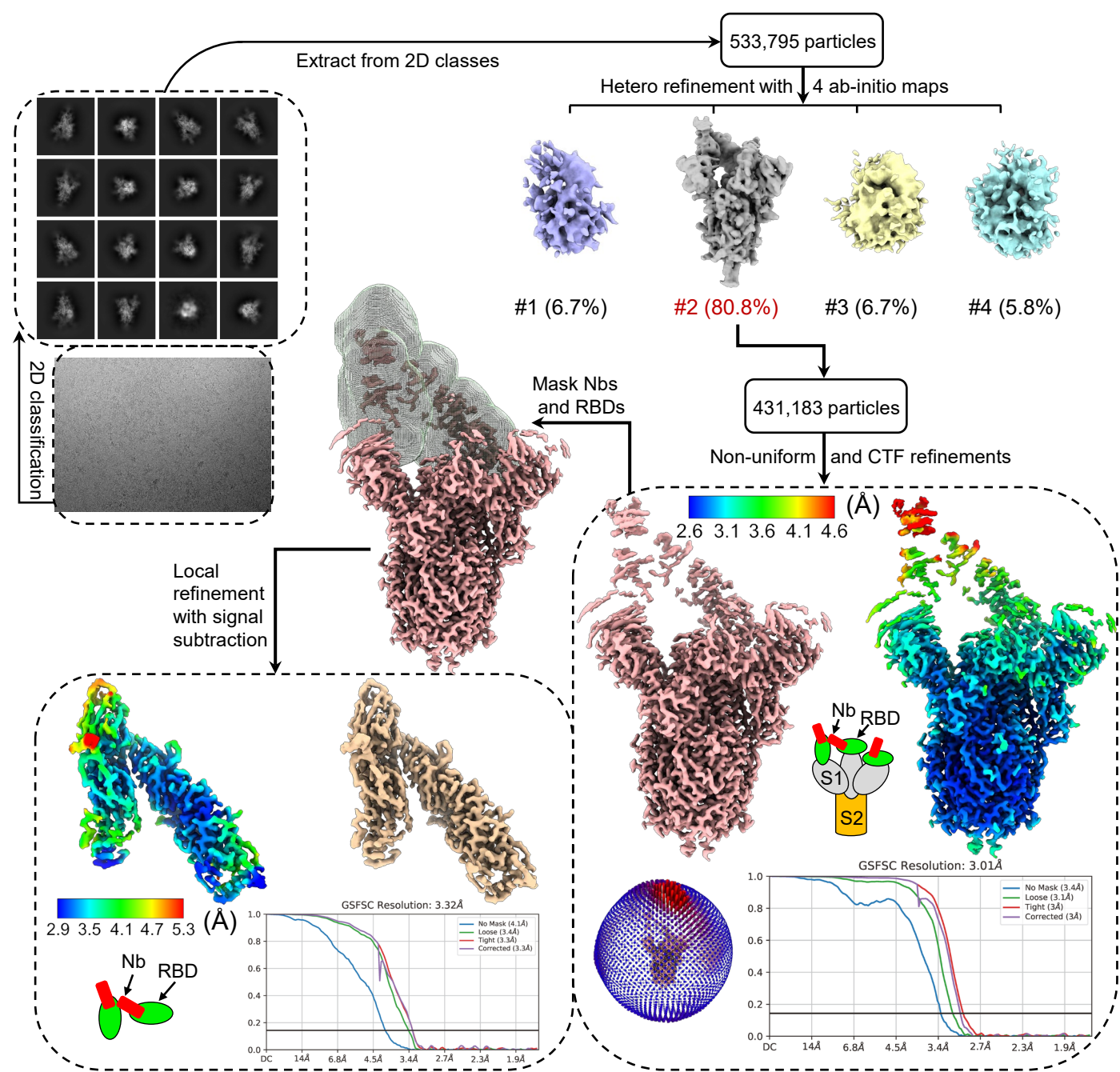

Supplement: S2 Fig — Representative raw cryo-EM image and 2D classes are presented. 3D refinements using all the particles from good 3D classes generated a 3.01 Å map. Further local refinement improved the density for the bound nanobody. The angular distribution plot, final maps, half-map FSC curves and accompanying local resolution illustrations are enclosed in the dashed black boxes. (PDF) [file ppat.1012726.s002.pdf]

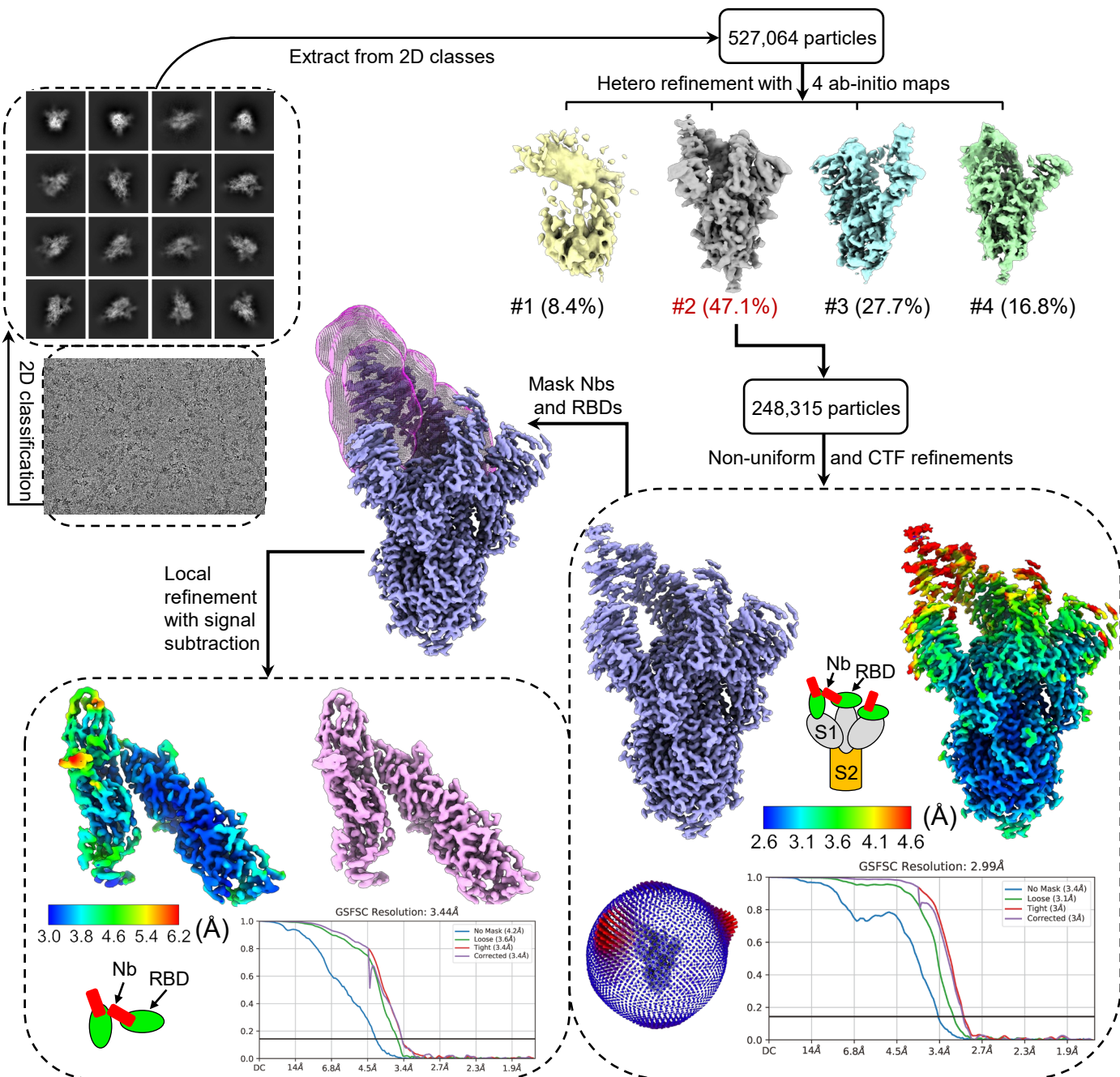

Supplement: S3 Fig — Representative raw cryo-EM image and 2D classes are presented. 3D refinements using all the particles from good 3D classes generated a 2.99 Å map. Further local refinement improved the density for the bound nanobody. The angular distribution plot, final maps, half-map FSC curves and accompanying local resolution illustrations are enclosed in the dashed black boxes. (PDF) [file ppat.1012726.s003.pdf]

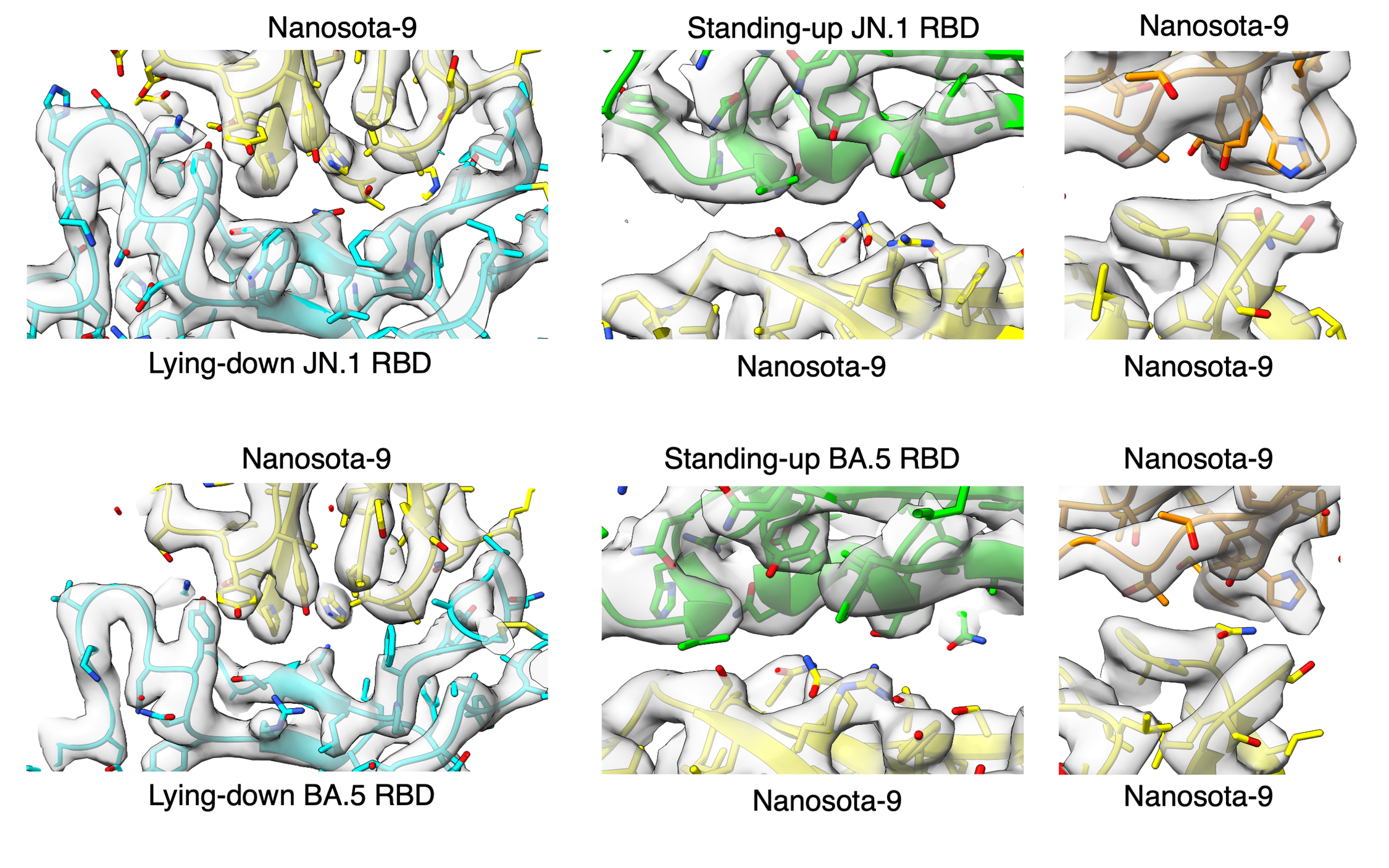

Supplement: S4 Fig — These three interfaces are: the major interface between the standing-up RBD and Nanosota-9, the minor interface between the lying-down RBD and Nanosota-9, and the additional interface between two Nanosota-9 molecules. (TIF) [file ppat.1012726.s004.tif]

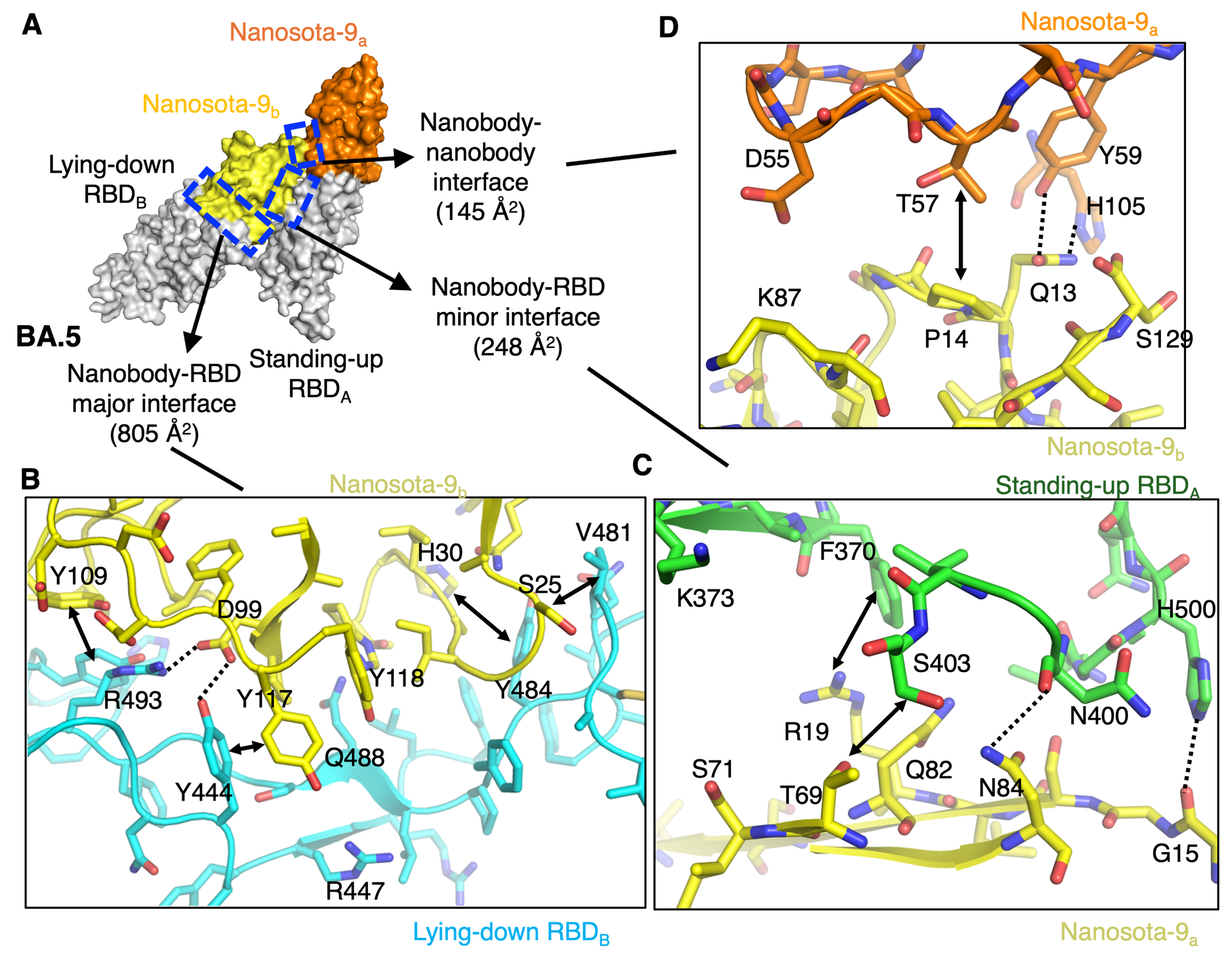

Supplement: S5 Fig — This figure was prepared in the same way as Fig 4, except that the BA.5 spike ectodomain was used instead of the JN.1 spike ectodomain. (TIF) [file ppat.1012726.s005.tif]

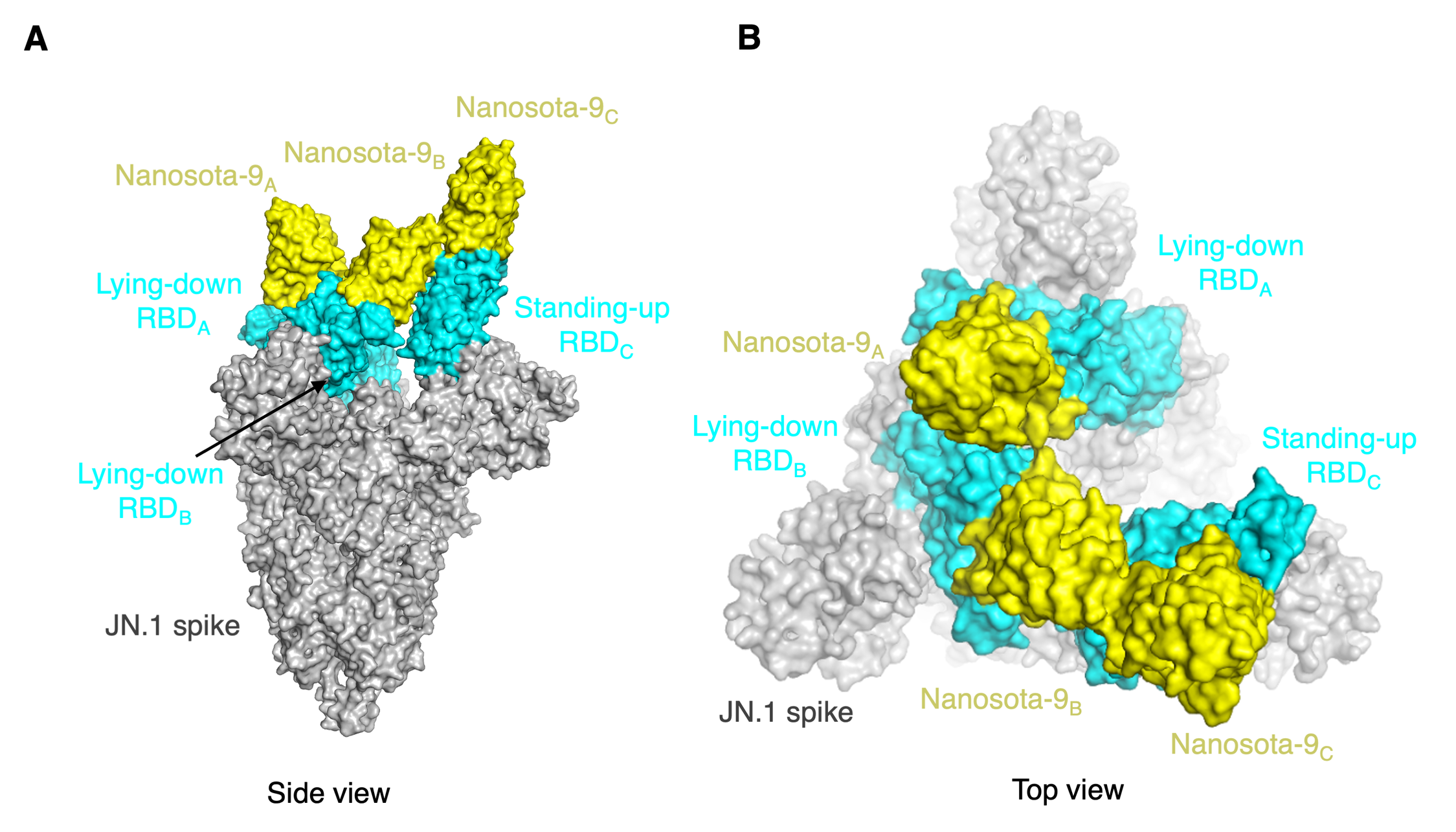

Supplement: S6 Fig — (A) Side view of the structure. (B) Top view of the structure. (TIF) [file ppat.1012726.s006.tif]

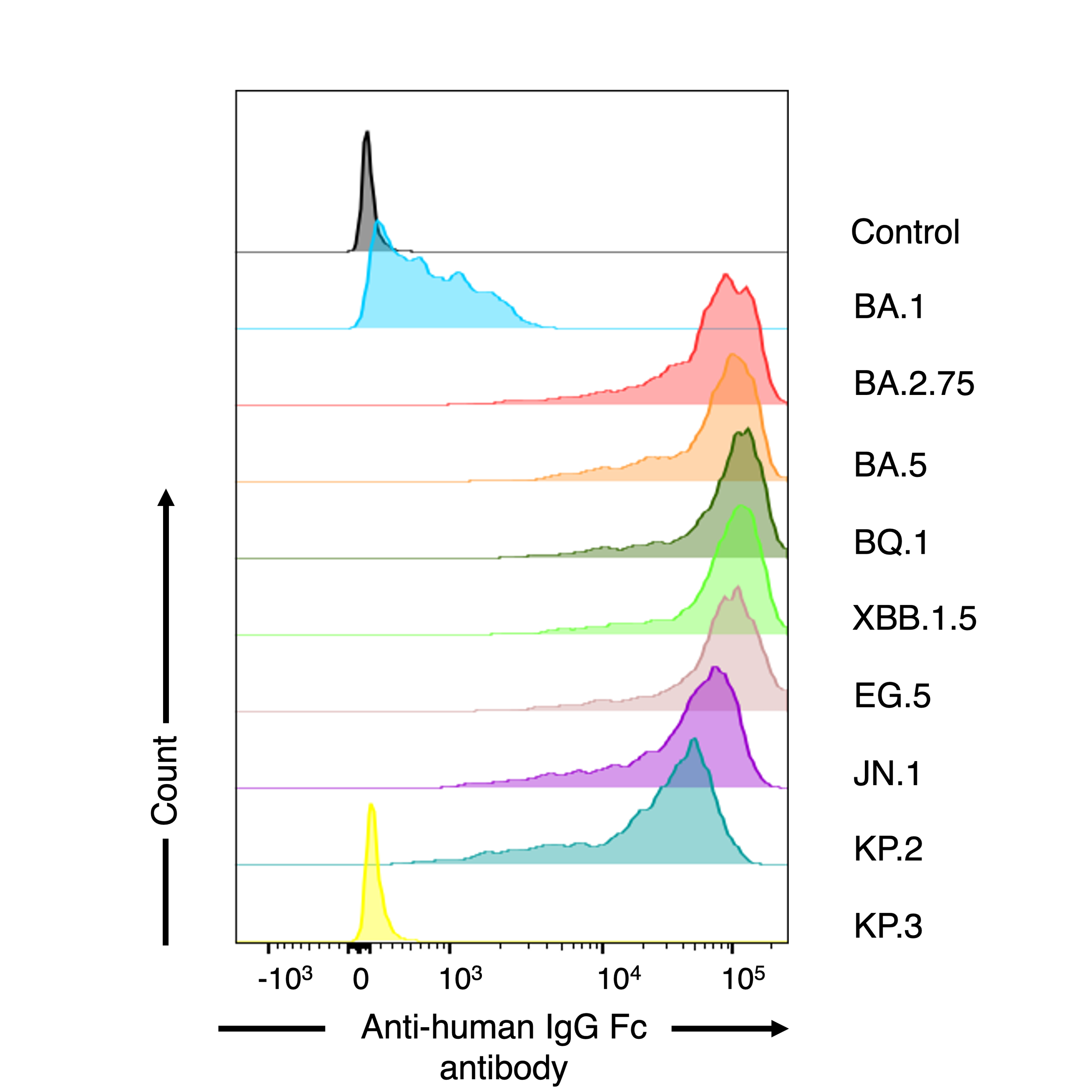

Supplement: S7 Fig — (TIF) [file ppat.1012726.s007.tif]

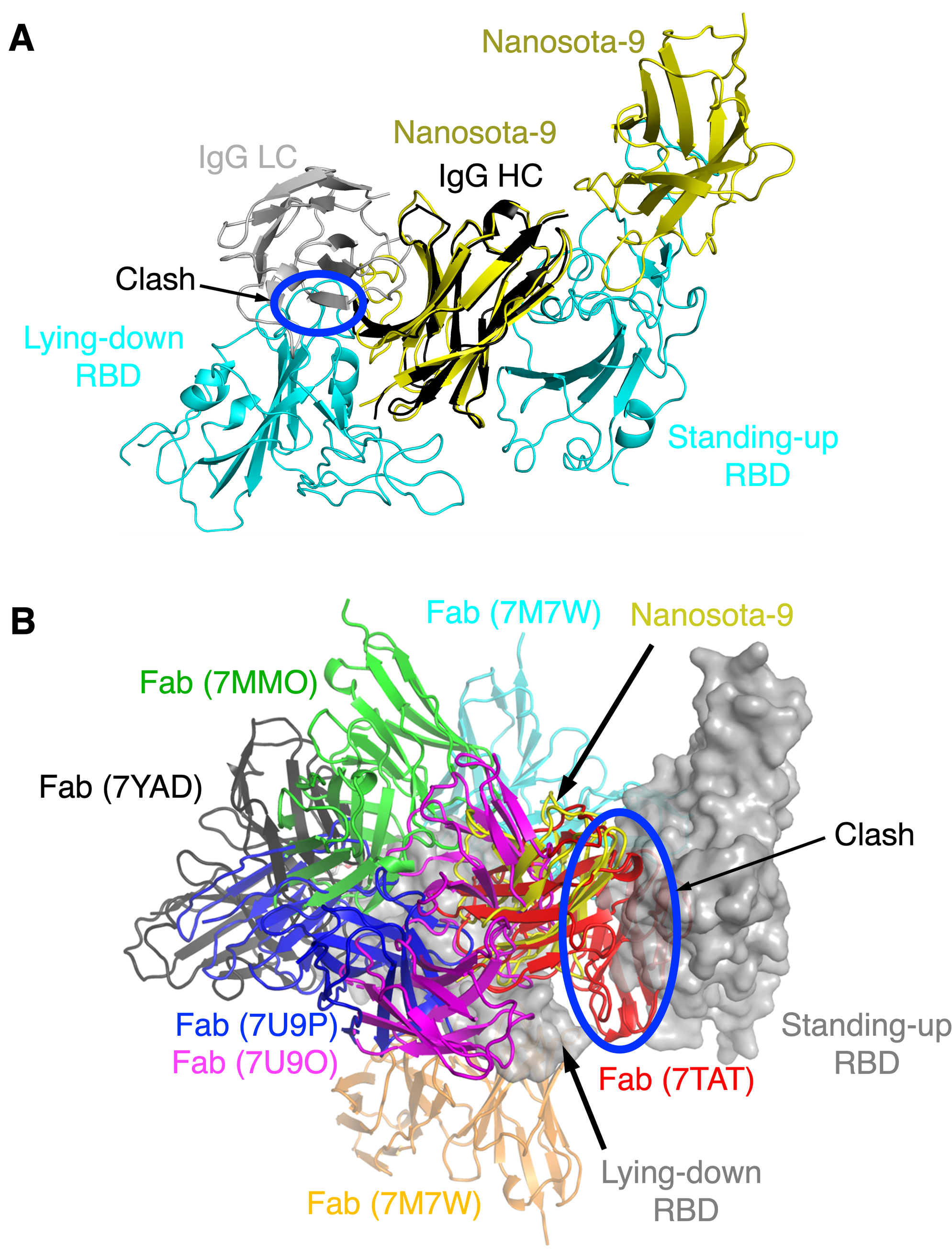

Supplement: S8 Fig — (A) Docking of a human antibody to the Nanosota-9 binding site on the Omicron spike. The antigen-binding domains of a human antibody (PDB 7B3O) were docked onto the structure of the JN.1 spike ectodomain/Nanosota-9 complex by structurally aligning the heavy-chain (HC) antigen-binding domain of the human antibody and Nanosota-9. Nanobodies and heavy-chain antigen-binding domains of human antibodies are evolutionarily and functionally related. The blue circle indicates a clash between the light-chain (LC) antigen-binding domain of the human antibody and the lying-down RBD, suggesting that human antibodies cannot access the Nanosota-9 binding site on Omicron spikes. (B) Overlay of the structures of human antibodies and Nanosota-9 all bound to the Omicron RBD. The structures of the Omicron spike ectodomains complexed with Fabs from human antibodies (PDB IDs labeled in parentheses) were overlaid with the JN.1 spike ectodomain/Nanosota-9 complex by structurally aligning their spike RBDs. Only one human antibody (PDB 7TAT) shares an overlapping epitope with Nanosota-9 on the lying-down RBD, but it clashes with a standing-up RBD, as indicated by the blue circle. Structural alignments were performed using PyMol. (TIF) [file ppat.1012726.s008.tif]

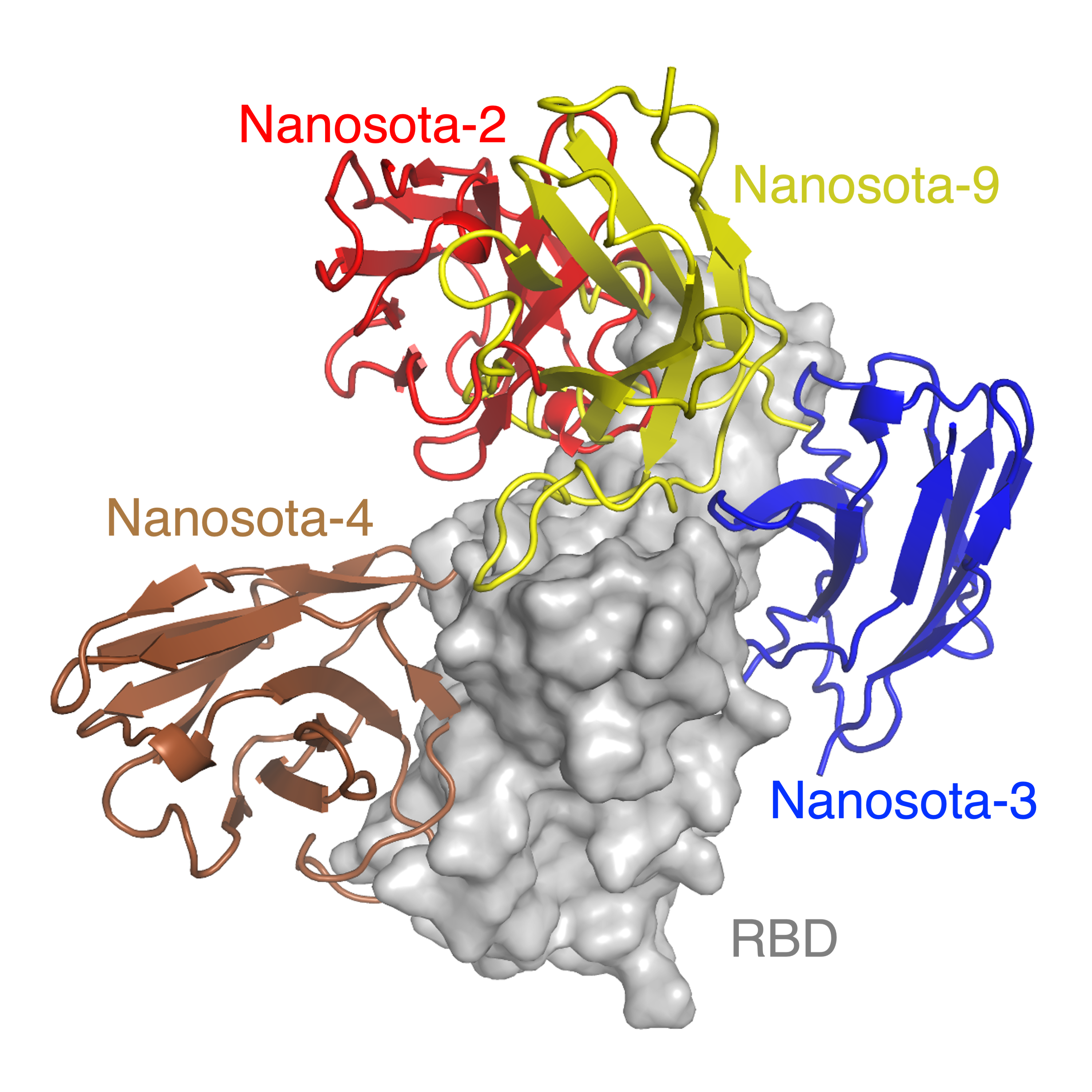

Supplement: S9 Fig — The structures of the prototypic SARS-CoV-2 spike each complexed with Nanosota-2, -3, or -4 (PDBs: 8G72, 8G74, and 8G75) were overlaid with the structure of the JN.1 spike complexed with Nanosota-9 through structural alignment of their spike RBDs. Nanosota-9 clashes with Nanosota-2 and -3, but not with Nanosota-4. Structural alignments were performed using PyMol. (TIF) [file ppat.1012726.s009.tif]
