# Supplementary material for: Discovery of Nanosota-9 as anti-Omicron nanobody therapeutic candidate
Source: PLoS Pathog. 2024 Nov 26;20(11):e1012726. doi: 10.1371/journal.ppat.1012726 (PMC11630572; doi:10.1371/journal.ppat.1012726)
Supplement: S1 Table — (PDF) [file ppat.1012726.s010.pdf]

Table S1: Cryo-EM data collection, refinement and validation statistics of the spike-Nanosota-9 complexes.

	BA.5 spike/ Nanosota-9 (EMDB-45771) (PDB 9CO6)	Local refinement of BA.5 spike/ Nanosota-9 with one RBD up and one RBD down (EMDB-45772) (PDB 9CO7)	JN.1 spike/ Nanosota-9 (EMDB-45773) (PDB 9CO8)	Local refinement of JN.1 spike/ Nanosota-9 with one RBD up and one RBD down (EMDB-45774) (PDB 9CO9)
**Data collection and processing**				
Magnification	130,000		130,000	
Voltage (kV)	300		300	
Electron exposure (e–/Å^2)	54.8		54.8	
Defocus range (μm)	-1.1 ~ -2.0		-1.1 ~ -2.0	
Pixel size (Å)	0.664		0.664	
Symmetry imposed	C1		C1	
Initial particle images (no.)	533,795	533,795	527,064	527,064
Final particle images (no.)	431,183	431,183	248,315	248,315
Map resolution (Å)	3.01	3.32	2.99	3.44
FSC threshold	0.143	0.143	0.143	0.143
Map resolution range (Å)	2.6–4.6	2.9-5.3	2.6–4.6	3.0-6.2
**Refinement**				
Initial model used (PDB code)	8IOS	8IOS	8IOS	8IOS
Model resolution (Å)	3.3	3.6	3.4	3.8
FSC threshold	0.5	0.5	0.5	0.5
Model resolution range (Å)	52.3-2.98	31.1-1.9	45.8-2.6	25.2-2.8
Map sharpening *B* factor (Å^2)	-102.1	-117.5	-94.2	-120.2
Model composition				
Non-hydrogen atoms	28152	5222	29029	5200
Protein residues	3546	666	3657	661
Ligands	36		32	
B factors (Å^2)				
Protein	149.64	152.92	54.19	70.26
Nucleotide				
Ligand	134.13		54.33	
R.m.s. deviations				
Bond lengths (Å)	0.004	0.005	0.005	0.006
Bond angles (°)	0.832	1.076	0.985	1.021
Validation				
MolProbity score	1.60	1.81	1.62	1.78
Clashscore	4.49	5.41	4.13	6.14
Poor rotamers (%)	0.03	0.18	0.03	0.72
Ramachandran plot				
Favored (%)	94.43	90.88	93.61	93.11
Allowed (%)	5.39	8.36	6.25	6.28
Disallowed (%)	0.17	0.76	0.14	0.61
